# Using GeoXAI to reveal gender differences in the spatially nonstationary influence of built environment on area-level happiness

**DOI:** 10.3389/fpubh.2026.1962036

**Published:** 2026-10-14

**Authors:** Cuicui Ye, Zheng Ding

**Affiliations:** 1 College of Art, Wuyi University, Wuyishan, Fujian, China; 2 College of Landscape Architecture and Art, Fujian Agriculture and Forestry University, Fuzhou, China

**Keywords:** area-level happiness, built environment, gender differences, GeoXAI, spatial differentiation, spatial nonstationarity

## Abstract

Gendered activity patterns can create unequal exposure to urban environments, yet city-scale evidence rarely combines gender-specific happiness, nonlinear modelling, and local spatial variation. We analysed 678,120 geotagged Weibo posts across 33,591 500 m grids in Shanghai using LSTM-CNN sentiment classification, HRNet streetscape segmentation, XGBoost, SHAP, partial dependence plots, and spatially varying contributions. After screening, 320,618 female-labelled and 187,089 male-labelled posts entered the analysis. Sentiment classification achieved 0.942 accuracy; random-fold XGBoost *R*^2^ was 0.898 and 0.930 for female- and male-labelled happiness, and remained positive under spatial separation and the 20-user threshold. Results showed: (1) positive expression accounted for 73.64% of female-labelled posts and 74.30% of male-labelled posts. Female-labelled grid scores were higher and more variable (4.06 ± 2.13 versus 2.94 ± 1.71); both surfaces were spatially clustered (Moran’s I = 0.909 and 0.666) but modestly correlated (*r* = 0.325; *ρ* = 0.273). (2) Population count and the Gini coefficient showed opposite correlations between groups. SHAP ranked NDVI first for the female-labelled model and Population count first for the male-labelled model, with Greenness, the Gini coefficient, and Distance to city center also prominent. (3) The same 5D dimension dominated only 43.3% of paired grids. Destination accessibility and Distance to Transit contributed more to female-labelled happiness in 95.1 and 84.0% of grids, whereas Design contributed more to male-labelled happiness in 68.0%. All five dimension-level differences were spatially clustered (Moran’s I = 0.571–0.788). These results locate contiguous gender differences in area-level happiness across Shanghai and provide evidence for locally targeted, gender-responsive urban and public-health planning.

## 1 Introduction

The built environment comprises the buildings, streets, transport networks, land uses, public spaces, and landscape elements that organize everyday urban activity (1, 2). Its density, functional mix, accessibility, street design, and transit connections shape the destinations people can reach, the routes they use, and the environments they encounter. Built-environment indicators account for 40 to 50% of the factors associated with residents’ expressed emotions (3). Emotion is a subjective response to environmental stimuli (4); it influences psychological states and social behaviour and is widely used to indicate urban happiness (5). Urban densification and continued built-environment expansion can also reduce emotional well-being when environmental burdens, limited access, or poor-quality public space accumulate (6–8). These relationships are gendered because daily mobility, care responsibilities, perceived safety, and access to urban resources differ between women and men. Examining gender differences in social-media-derived area-level happiness can therefore sharpen place-specific urban renewal and support Sustainable Development Goals 3 and 11 (9).

Stock-oriented urban renewal has become an important strategy for advancing urban sustainability (10). Research on the built environment and residents’ emotions has generally adopted a broad perspective, examining the roles of urban liveability (10), urban functions (11, 12), urban vitality (13, 14), art in public space (15), and landscape perception (16, 17). This work has established an important basis for understanding the benefits of the built environment. However, its complexity and diversity have limited knowledge of spatial nonstationarity. Traditional studies have measured emotional responses through electroencephalography (18), online surveys (19), questionnaires (20), and psychophysiological experiments (16). These methods can capture subtle responses, but their long data-collection cycles and limited spatial coverage constrain city-scale analysis in rapidly changing environments. Emotional responses also differ by gender (21–23), and the relationship between environment and emotion is rarely linear. Consequently, existing approaches have difficulty resolving fine-grained gender differences in happiness and their spatial variation across large cities.

Urban big data, machine learning, and social media have broadened the methodological scope of emotion research. User-generated content (UGC) and machine learning are increasingly used to examine relationships between the built environment and residents’ emotions (24). Applications include characterizing city image from textual sentiment (25), integrating machine learning with conventional surveys (26), quantifying heritage value from UGC (27), and measuring regional happiness through media-sentiment indices (28). Consistent with this literature, we use social-media-derived area-level happiness (hereafter, area-level happiness) to denote the mean sentiment-derived happiness score expressed within each 500 m grid. This ecological construct characterizes the affective tone associated with an urban area on a common spatial support. Privacy restrictions and incomplete profile information nevertheless limit access to user attributes. Most city-scale studies therefore emphasize average responses among residents or visitors and provide limited insight into fine-grained gender differences. Natural language processing methods such as random forests (29), transformer-based deep learning (30), DeepSentiBank (31), and Inception-v3 (32) have also been applied to affective data. Their results can be affected by subjective labelling, contextual dependence, and computational cost (33). Spatial statistical models rely on assumptions that may be restrictive when the data-generating process is complex, while conventional spatial modelling can miss nonlinearity and interactions. Machine-learning models require fewer prior assumptions but remain difficult to interpret (34). This study therefore combines gender-specific UGC, multi-source urban data, and explainable spatial machine learning to identify gender differences in area-level happiness and their spatial nonstationarity.

Shanghai combines very high density, extensive public transport, marked centre-periphery differences, and a heterogeneous mix of historic, renewed, and rapidly urbanizing districts. Its gross domestic product has remained among the highest of Chinese cities (35), and sustained migration has consolidated its role as a national economic centre (36). Rapid urbanization has expanded construction land, population, energy consumption, and motor-vehicle use (37). National policies have simultaneously promoted public services, urban greenery, and functional renewal (38). The resulting contrasts in density, accessibility, streetscape quality, and socioeconomic context make Shanghai a demanding setting in which to test nonlinear and spatially varying built-environment associations (39). Geographically uneven relationships with emotion and health further support a gender-specific analysis of area-level happiness in this high-density city (40).

Using Shanghai as a case study, we examine nonlinear and spatially nonstationary associations between the built environment and female- and male-labelled area-level happiness. Three gaps guide the analysis. First, city-scale research rarely evaluates gender differences in area-level happiness together with nonlinear environmental associations and local spatial variation (22, 41–45). Second, common-grid analyses often overlook spatial support, streetscape coverage, and the loss of predictive transferability across geographically separated areas (46–49). Third, high-resolution evidence remains limited on how female and male happiness surfaces and their environmental attributions differ across the same urban locations (50–52). We address these gaps by integrating geotagged UGC, sentiment classification, HRNet streetscape segmentation, and XGBoost; comparing alternative models under identical random and buffered spatial folds; testing minimum-user and streetscape-support sensitivity; and combining out-of-fold SHAP, full-variable partial dependence, spatially varying contributions, and cross-surface spatial diagnostics. This GeoXAI design distinguishes citywide nonlinear associations from local environmental contributions and identifies spatially contiguous gender differences in area-level happiness.

Beyond Shanghai, the design can be applied where geotagged public expression and multi-source environmental data are available. Local recalibration and spatial validation define its use in each city.

## 2 Materials and methods

### 2.1 Study area

Shanghai is one of China’s most developed cities, with a permanent population of 24.8 million across 16 administrative districts. It lies on China’s eastern coast between 120 degrees 52 min and 122 degrees 12 min E and between 30 degrees 40 min and 31 degrees 53 min N. The city has a mean elevation of 2.19 m and a total area of 6340.5 km^2^. Economic growth, rapid urbanization, and land-use restructuring have produced pronounced internal differentiation and increased spatial heterogeneity in the built environment (53). Shanghai is therefore a suitable case for examining gender differences in area-level happiness. Figure 1 presents the study area, including green space, built-up land, and water. A 500 m fishnet covering the municipality was created in ArcMap 10.8 to align the study variables while retaining intra-urban spatial detail.

**Figure 1:**
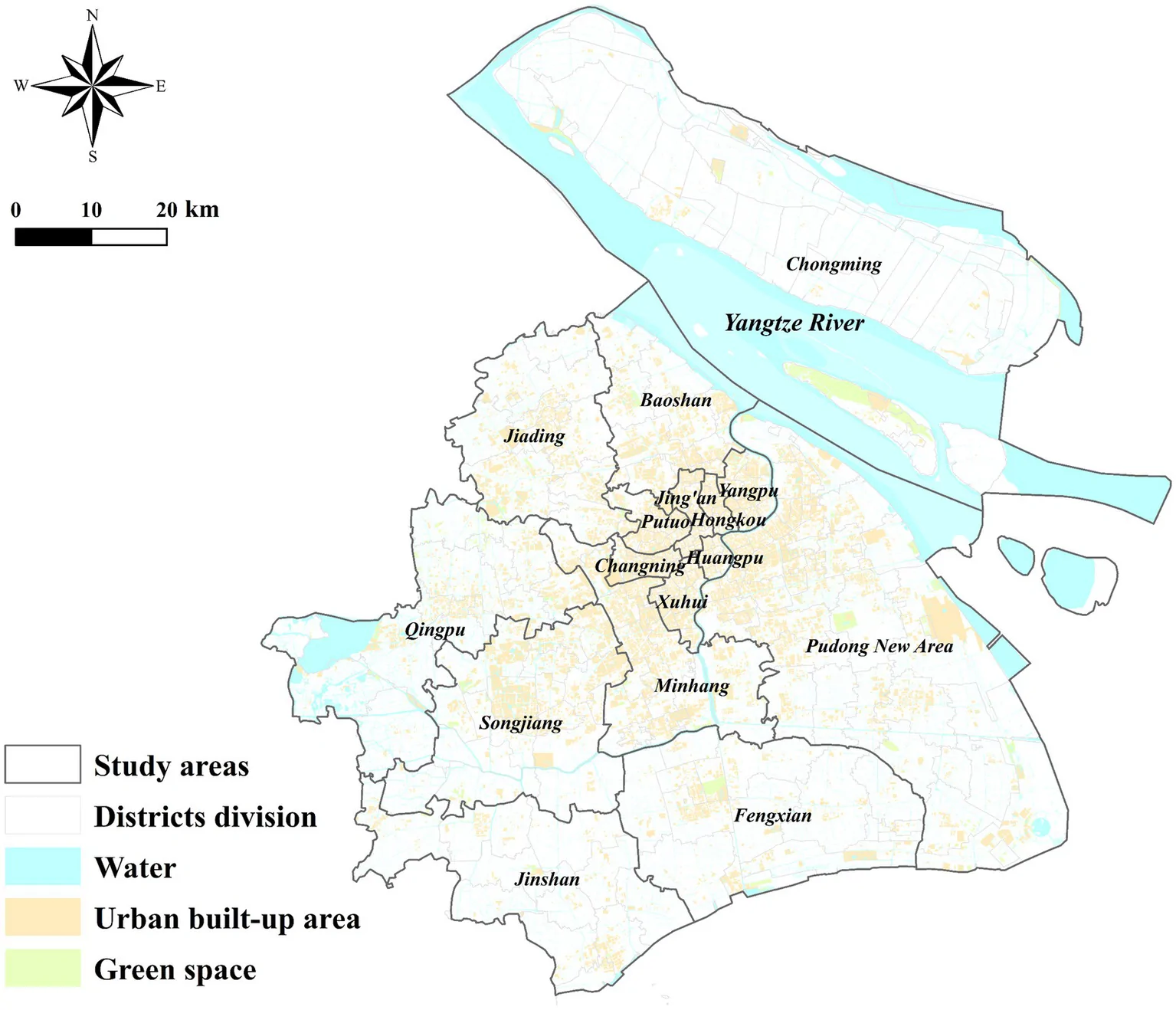
Study area and analytical grid in Shanghai.

### 2.2 Technical route

We combined ERNIE-LSTM-TextCNN, HRNet, and XGBoost to examine how the built environment was associated with female and male area-level happiness. The workflow comprised UGC acquisition, sentiment analysis, built-environment measurement, correlation and spatial-autocorrelation analysis, and explainable spatial machine learning. ArcGIS 10.8 was used for spatial matching and visualization. The four stages were data collection, indicator calculation, data analysis, and interpretation (Figure 2).

**Figure 2:**
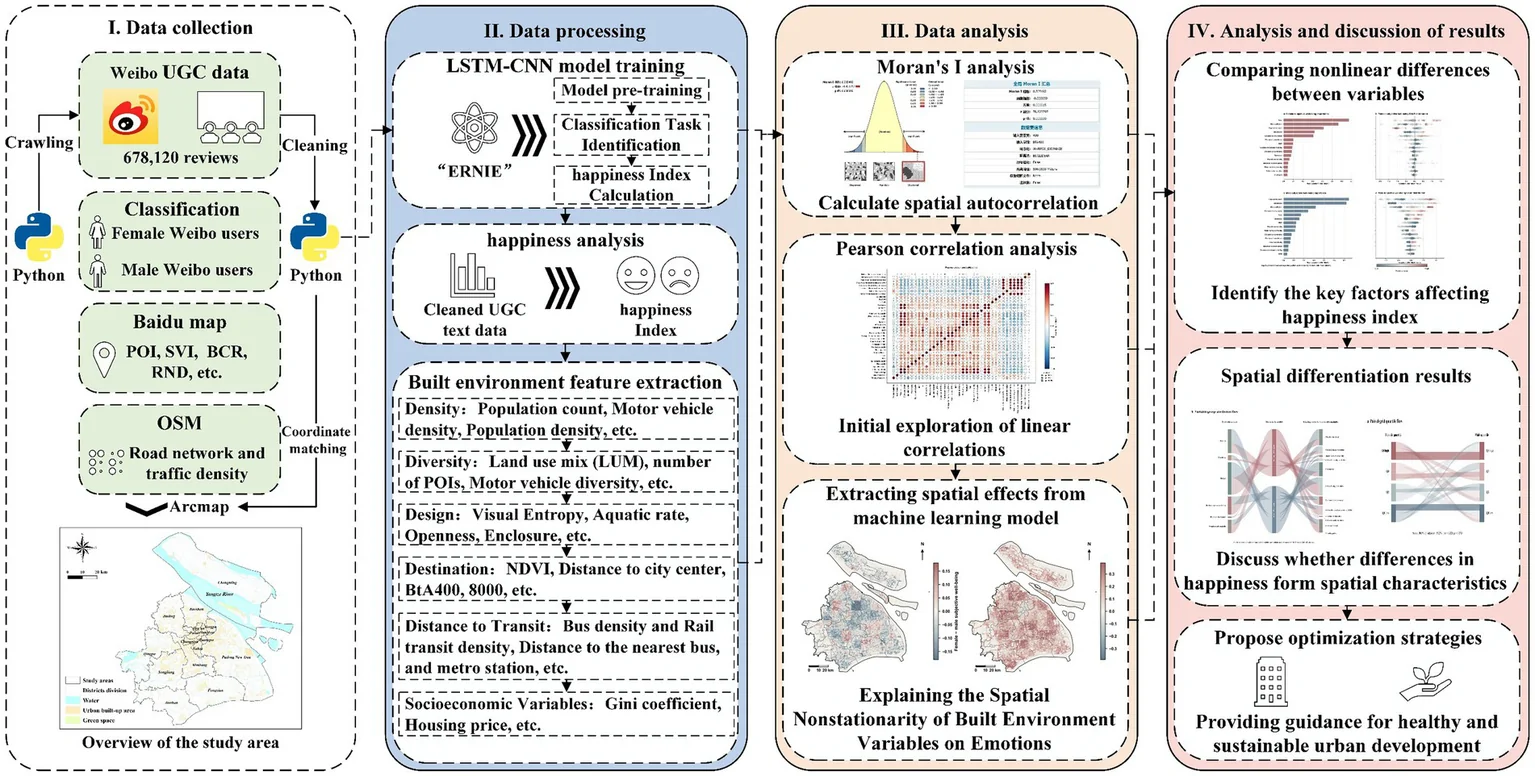
Technical route.

#### 2.2.1 Data collection

A Python workflow retrieved 678,120 publicly visible, geotagged Weibo records in the study area from 1 November 2024 to 1 May 2025. The final polarity-scored corpus contained 507,707 records: 320,618 with a female platform label and 187,089 with a male platform label. Baidu Maps, OpenStreetMap, Baidu Street View, remote sensing, population, and housing-price data described population, road networks, accessibility, green landscapes, streetscape perception, and socioeconomic context. Coordinates and projections were harmonized in ArcGIS 10.8.

#### 2.2.2 Indicator calculation

The ERNIE-LSTM-TextCNN model estimated a sentiment index for each UGC text. Female and male area-level happiness scores were then aggregated separately within 500 m grids. Built-environment indicators were organized according to the 5D framework. ArcGIS was used to calculate density, functional diversity, destination accessibility, and transit distance. HRNet and MATLAB derived streetscape indicators, including Greenness, Openness, Enclosure, PavingDegree, Visual entropy, and Color complexity.

#### 2.2.3 Data analysis

Pearson correlations and Global Moran’s I described linear associations and spatial patterns. Separate XGBoost models were fitted for female- and male-labelled area-level happiness. Model development compared XGBoost with Ridge regression, partial least squares (PLS) regression, K-nearest neighbours (KNN), and linear support vector regression (Linear SVR), while geographic transfer was evaluated with random and buffered spatial folds on the same 19,113 grids and 30 predictors. Minimum-user and streetscape-support sensitivity analyses evaluated sample support. Test-set SHAP values, 5D attribution, full-variable partial dependence plots (PDPs), and out-of-fold spatially varying contributions (SVCs) quantified overall model attribution, nonlinear relationships, and local spatial differences.

#### 2.2.4 Interpretation

We compared the spatial distributions, predictive performance, influential variables, nonlinear ranges, and spatial nonstationarity of the two area-level happiness outcomes. The findings were interpreted in relation to built-environment research, gendered mobility and exposure, and planning strategies for healthier and more equitable cities.

### 2.3 Indicator selection

The indicator system followed the 5D framework proposed by Ewing and Cervero: Density, Diversity, Design, Destination accessibility, and Distance to Transit (54). Socioeconomic variables were added to represent housing costs and broader resource distribution. Table 1 summarizes the research elements and indicator composition. Definitions, source websites where available, acquisition details, processing, spatial support, and missing-value treatment for all 36 predictors and the two outcomes are reported in Supplementary Table S1.

**Table 1:** Research element breakdown.

Research element	Indicator composition	Primary quantification method	Analytical role	Detailed variables
Resident happiness	Female and male area-level happiness	ERNIE-LSTM-TextCNN	Outcomes	Supplementary Table S1
Density	Six density indicators	HRNet, GIS	5D dimension	Supplementary Table S1
Diversity	Ten functional- and visual-diversity indicators	HRNet, MATLAB, GIS	5D dimension	Supplementary Table S1
Design	Six streetscape and vegetation indicators	HRNet, GEE, GIS	5D dimension	Supplementary Table S1
Destination accessibility	Eight centrality and destination-distance indicators	sDNA and GIS network analysis	5D dimension	Supplementary Table S1
Distance to Transit	Four public-transport supply and distance indicators	GIS network analysis	5D dimension	Supplementary Table S1
Socioeconomic variables	Gini coefficient、Housing price	GIS and contextual data	Contextual controls	Supplementary Table S1

#### 2.3.1 Density

Density captures built intensity and the concentration of people, traffic, and road capacity. Building coverage ratio (BCR) and floor area ratio (FAR) represent built intensity, whereas Population density, Population count, Motor vehicle density, and road network density (RND) represent population, traffic activity, and road-network capacity (55, 56).

#### 2.3.2 Diversity

This dimension describes the functional mix of land use, facility types, and the heterogeneity of visual streetscape information. Indicators include land-use mix, six categories of points of interest (POIs), Motor vehicle diversity, Visual entropy, and Color complexity. Higher entropy indicates greater functional or visual diversity (57, 58). Color complexity entered the 36-variable main model but was excluded from the strict spatial model by fold-specific variance inflation factor screening.

#### 2.3.3 Design

This dimension captures street-network form and pedestrian-scale environmental perception through Greenness, Openness, Enclosure, PavingDegree, Aquatic rate, and the normalized difference vegetation index (NDVI) (59, 60). NDVI represents broader vegetative context, whereas Greenness represents visible street-level vegetation. Both entered the model at their observed spatial supports. No difference, absolute-difference, or product terms were constructed from the two measures.

#### 2.3.4 Destination accessibility

This dimension measures connections to the city centre and to residential, commercial, green-space, industrial, and transport destinations. It also includes BtA400 and BtA8000, calculated with spatial Design Network Analysis (sDNA), to represent network betweenness at different radii (54, 61–63). These measures indicate spatial proximity and potential travel cost; observed trips and facility use were outside the input data.

#### 2.3.5 Distance to transit

This dimension comprises bus-route density, rail-transit density, and network distances to the nearest bus and metro stations. Together, these variables describe public-transport supply, proximity, and first- and last-mile travel costs (64, 65).

#### 2.3.6 Socioeconomic variables

Because built-environment perception also reflects the distribution of urban resources and housing costs, the Gini coefficient and Housing price were included as contextual variables (63, 66–69). Both entered the 36-variable main model. Housing price was retained in the strict spatial model. The Gini coefficient had only 17 area-level values and was therefore excluded from the strict 500 m PDP and SVC analysis.

### 2.4 Data collection

#### 2.4.1 Social media data

Geotagged social-media data have been used to measure expressed happiness and urban sentiment across parks, cities, and broader urban systems (5, 70–72). In this study, area-level happiness is operationalized as the mean sentiment-derived score of publicly visible, geotagged posts within each 500 m grid, calculated separately for the female and male platform-label groups. Using Python 3.9 and publicly accessible Weibo mobile-location pages, we retrieved 678,120 geotagged records in Shanghai from 1 November 2024 to 1 May 2025. The raw file contained 18,948 records without a public platform-gender label, 152 records without text, and no missing longitude or latitude values; these conditions could overlap. Records without a public platform-gender label or usable text were excluded. Before sentiment scoring, posts shorter than 5 characters or longer than 150 characters, together with posts composed solely or predominantly of emojis or special characters and therefore lacking interpretable subjective affective content, were treated as invalid text and excluded. URLs and hashtags were removed, emojis and other non-text symbols were stripped from retained posts, and exact repeated normalized texts were deleted before scoring. These steps retained 659,067 records before text normalization, 659,064 after normalization, and 590,513 after exact cleaned-text deduplication. Polarity scoring was completed for 507,707 records: 320,618 female-labelled and 187,089 male-labelled posts. Supplementary Table S9 reports the processing counts. The fixed six-month window improved temporal comparability across grids (73, 74).

The analytical table contained female- and male-labelled area-level happiness values for 33,591 500 m grids (41). A separate post- and user-count file matched 507,550 scored records (99.97% of the scored corpus), comprising 320,552 female-labelled and 186,998 male-labelled posts across 7,252 grids. The remaining 157 scored records fell just outside Shanghai’s municipal boundary after coordinate alignment and therefore could not be assigned to a 500 m grid. This file was used to describe sampling support and define the minimum-user subsets, while the outcome columns in the final analytical table were retained for modelling. Among the represented grids, 4,832 contained at least one user from each group. Median support was four female-labelled and five male-labelled posts, with a median of three unique users in each group. Table 2 reports district-level post composition, and Supplementary Tables S9, S10 report post and user support.

**Table 2:** District-level composition of matched platform-label posts and Seventh National Population Census counts.

District	Male-labelled posts, *n* (%)	Female-labelled posts, *n* (%)	Male census population	Female census population
Chongming	1,370 (48%)	1,504 (52%)	334,447 (52%)	303,474 (48%)
Fengxian	1,570 (51%)	1,497 (49%)	614,601 (54%)	526,271 (46%)
Hongkou	8,101 (35%)	15,309 (65%)	373,314 (49%)	384,184 (51%)
Huangpu	23,405 (27%)	63,520 (73%)	343,122 (52%)	318,908 (48%)
Jiading	6,244 (42%)	8,733 (58%)	988,329 (54%)	845,929 (46%)
Jinshan	1,361 (62%)	834 (38%)	437,838 (53%)	384,938 (47%)
Jing’an	13,330 (32%)	27,956 (68%)	475,832 (49%)	499,875 (51%)
Minhang	19,793 (53%)	17,768 (47%)	1,376,024 (52%)	1,277,465 (48%)
Pudong New Area	50,916 (39%)	80,706 (61%)	2,950,524 (52%)	2,730,988 (48%)
Qingpu	8,980 (51%)	8,634 (49%)	694,529 (55%)	576,895 (45%)
Songjiang	6,410 (42%)	8,756 (58%)	1,020,220 (53%)	889,493 (47%)
Xuhui	14,957 (31%)	33,281 (69%)	542,439 (49%)	570,639 (51%)
Yangpu	9,449 (47%)	10,837 (53%)	614,720 (49%)	627,828 (51%)
Changning	8,012 (28%)	20,526 (72%)	330,033 (48%)	363,018 (52%)
Putuo	6,648 (36%)	11,898 (64%)	610,921 (49%)	628,879 (51%)
Baoshan	6,452 (42%)	8,793 (58%)	1,168,318 (52%)	1,066,900 (48%)

#### 2.4.2 Multi-source urban data

We integrated Baidu Maps POIs and building areas of interest (AOIs), OpenStreetMap roads, Baidu Street View imagery, remote-sensing vegetation, population, housing-price, and socioeconomic data. All datasets were projected to a common coordinate system and joined or aggregated to the 500 m grid. Supplementary Table S1 reports source websites, acquisition details, processing, missing-value rules, and native and analytical spatial supports.

(1) POI and building AOI data were obtained from Baidu Maps. Coordinates, categories, and building footprints were used to calculate six POI densities, BCR, FAR, and distances to destinations.
(2) Road-network data were obtained from OpenStreetMap. They were used to calculate RND, bus- and rail-network density, sDNA centrality, and network distances to public-transport stations and other destinations.
(3) Street-view images were acquired through the Baidu Street View interface in March 2025. Sampling points were placed every 50 m along the road network. Images were collected at azimuths of 0, 90, 180, and 270 degrees at each available point. After quality screening, 1,887,356 images were retained to derive streetscape indicators within the Density, Diversity, and Design dimensions.
(4) Population density, Population count, the Gini coefficient, and Housing price were obtained from population and socioeconomic data layers. Population count and the Gini coefficient each contained 17 area-level values in the final analytical table. Housing price retained 500 m grid support.
(5) NDVI was derived from nominal 30 m imagery in Google Earth Engine and aligned with the 500 m analytical grid. The final table contained only 17 area-level NDVI values. It was therefore interpreted according to its observed support rather than its nominal remote-sensing resolution.

### 2.5 Data processing and analysis

All UGC and multi-source urban data were aligned to a 500 m analytical grid. The main dataset comprised 33,591 grids, two outcomes for female- and male-labelled area-level happiness, and 36 built-environment and contextual predictors listed in Supplementary Table S1. Both outcome models used the same grid definitions, parameters, and validation procedures. The de-identified analytical data are provided as Supplementary Data S1, the PDP and SVC summaries as Supplementary Data S2, and the validation and sensitivity tables, out-of-fold predictions, and reproducibility manifest as Supplementary Data S3.

Minimum-user sensitivity retained grids in which both platform-label groups had at least 1, 3, 5, 10, or 20 unique users. The final happiness scores were held fixed, and XGBoost used the same random five-fold procedure at each threshold. Among the 33,591 grids, 14,478 had zero values for all eight streetscape indicators. The streetscape-supported analysis used the remaining 19,113 grids and 30 predictors. Population count, the Gini coefficient, and NDVI were excluded because their realised support was regional; Color complexity, BCR, and Commercial POIs were excluded by fold-specific variance inflation factor screening. Sensitivity to streetscape support was assessed with a matched 23-predictor non-streetscape model on the same 19,113 grids and on all 33,591 grids.

#### 2.5.1 ERNIE-LSTM-TextCNN sentiment analysis

The ERNIE-LSTM-TextCNN framework combines long short-term memory representations of sequential context with convolutional extraction of local affective features (75). ERNIE generated contextualized text representations, a unidirectional LSTM encoded sequence information, and a TextCNN branch extracted local features. The model classified positive and negative expression and transformed post-level outputs to a continuous score from −10 to 10, with higher values indicating more positive expression. These group-specific scores define the area-level happiness variables in the final 500 m analytical table.

Model evaluation used 12,187 labelled records after excluding training-corpus overlap, duplicates, and conflicting labels. The ERNIE-LSTM-TextCNN classifier was compared on the same holdout with character TF-IDF Multinomial Naive Bayes, character-count Bernoulli Naive Bayes, and a training-derived log-odds sentiment lexicon. These conventional baselines represent probabilistic bag-of-character and lexicon-based sentiment classification. Accuracy, balanced accuracy, macro-F1, weighted F1, Matthews correlation, and confusion matrices were reported (Supplementary Table S11).

#### 2.5.2 HRNet streetscape semantic segmentation

HRNet was used to extract landscape elements from the street-view images. Its parallel high- and low-resolution representations preserve spatial detail through repeated feature fusion (76). On the Cityscapes test set, the reported mean intersection over union was 81.6%, instance-level intersection over union was 61.8%, category intersection over union was 92.1%, and category-level instance intersection over union was 82.2%. We used HRNet to identify vegetation, sky, water, paved surfaces, enclosing elements, and motor vehicles. These classes were converted into Greenness, Openness, Enclosure, PavingDegree, Aquatic rate, Motor vehicle density, and Motor vehicle diversity using the definitions in Supplementary Table S1 (60).

#### 2.5.3 Visual entropy and color complexity

Visual entropy measures the overall information complexity of a street-view image. Images were enhanced, segmented, and summarized in MATLAB, and entropy was calculated from the occurrence probabilities of segmented regions. Higher values indicate richer visual information. Color complexity was calculated from color distributions, connected regions, and pixel extent. Both variables entered the 36-variable main model and were assigned to Diversity. Color complexity was excluded from the strict spatial model by fold-specific variance inflation factor screening (57, 58).

#### 2.5.4 Correlation and spatial autocorrelation

Pearson correlations summarized unadjusted linear associations between the two happiness outcomes and the built-environment variables. The matrix served solely as a descriptive summary. Global Moran’s I (Equation 1) was used to assess spatial autocorrelation in the observed happiness surfaces and coordinate-grouped out-of-fold residuals (77):

$$I=\frac{n}{\sum_{i}\sum_{j}w_{\mathrm{ij}}}\frac{\sum_{i}\sum_{j}w_{\mathrm{ij}}(x_{i}-\bar{x})(x_{j}-\bar{x})}{\sum_{i}{\left(x_{i}-\bar{x}\right)}^{2}}$$ (1)

where n is the number of grids, x_i is the observed value in grid i, x̄ is the sample mean, and w_ij is the spatial weight. Binary Rook contiguity was constructed from the 500 m analytical grid. A weight of 1 was assigned only to grids sharing a boundary segment of positive length, producing 66,378 undirected adjacencies and no islands. Global Moran’s I was evaluated using 999 two-sided permutations.

#### 2.5.5 XGBoost prediction, geographic validation, and SHAP analysis

Separate XGBoost regression models examined nonlinear associations between the built environment and female- and male-labelled area-level happiness, and SHAP provided model attribution (34, 42–44). The citywide analysis used all 33,591 grids and the 36 original predictors, without engineered features. XGBoost used 500 trees, maximum depth 5, learning rate 0.04, subsample 0.90, column sampling 0.85, L1 regularization 0.05, L2 regularization 1.0, squared-error loss, and seed 42.

Citywide predictive performance was assessed using random five-fold and coordinate-grouped five-fold out-of-fold validation. A stricter transfer analysis used the 19,113 streetscape-supported grids and 30 predictors, assigned 10 km spatial blocks to five outer folds, removed training observations within 2.5 km of each test block, and selected XGBoost parameters by inner-fold tuning within each outer training set. Each out-of-fold prediction was generated exactly once, and outer test grids did not enter parameter selection. Table 3 reports these three XGBoost validation settings.

**Table 3:** XGBoost performance under three out-of-fold validation settings.

Outcome and validation	*R*^2^ (95% CI)	RMSE	MAE
Male-labelled; random five-fold	0.930 (0.928–0.933)	0.451	0.214
Female-labelled; random five-fold	0.898 (0.893–0.902)	0.681	0.306
Male-labelled; coordinate-grouped five-fold	0.882 (0.879–0.886)	0.586	0.320
Female-labelled; coordinate-grouped five-fold	0.759 (0.750–0.767)	1.046	0.511
Male-labelled; strict spatial transfer	0.371	1.102	0.879
Female-labelled; strict spatial transfer	0.356	1.808	1.486

Random and coordinate-grouped analyses used all 33,591 grids and 36 original predictors with the fixed XGBoost specification. Strict spatial transfer used 19,113 streetscape-supported grids and 30 predictors, with hyperparameters selected within each outer training fold; its values are point estimates. The matched conventional-model comparison is reported in Table S12.

The model-development comparison used the same 19,113 grids, 30 predictors, and random and buffered folds for Ridge regression, PLS regression, KNN, Linear SVR, and XGBoost. The four conventional models were standardized within each training fold. Ridge used an alpha of 10; PLS used 10 latent components with internal scaling disabled; KNN used 25 distance-weighted Euclidean neighbours; and Linear SVR used C = 1.0, epsilon = 0.1, and squared epsilon-insensitive loss. XGBoost used the fixed specification reported above. Performance was reported as pooled out-of-fold *R*^2^, RMSE, and MAE. Test grids were excluded from fitting and preprocessing. Full results, model settings, fold specifications, and out-of-fold predictions are provided in Supplementary Tables S12–S14 and Supplementary Data S3.

Within each random fold, TreeSHAP explained only test grids that were not used for model training. Test-set SHAP values were pooled across folds, and mean absolute SHAP values summarized each predictor’s contribution to model predictions. SHAP values are interpreted as model attributions under the observed predictor distribution. The additive explanation is defined as follows (Equation 2):

$$g(z^{\prime })=\phi_{0}+\sum_{i=1}^{M}\phi_{i}z\prime_{i}$$ (2)

where g(z′) is the model prediction, φ₀ is the expected prediction, M is the number of predictors, and φᵢ is the SHAP attribution of predictor i. Spearman correlations between fold-specific rankings and the pooled ranking were also calculated to assess the stability of variable importance.

#### 2.5.6 Out-of-fold spatially varying contributions

Out-of-fold SVCs were calculated within the strict spatial model using 19,113 grids and 30 predictors to estimate local model contributions (45). For each outer-fold test grid, a target predictor was set in turn to the 25th and 75th percentiles of the corresponding training fold while all other predictors retained their observed values. The difference between the two predictions defined the SVC (Equation 3):

$${\mathrm{SVC}}_{\text{if}}={\hat{y}}_{\text{if}}(Q_{75,f})-{\hat{y}}_{\text{if}}(Q_{25,f})$$ (3)

If the 25th and 75th percentiles were identical, the contrast was expanded symmetrically in one-percentile increments until the nearest distinct empirical values were found. This rule was required only for Rail transit density and Green space (POIs), and the contrast values were fixed before evaluating the test fold. A positive SVC indicates that the prediction increased from the low to the high contrast value; a negative SVC indicates that it decreased. SVC is interpreted as a local contrast in the out-of-fold fitted function.

#### 2.5.7 Spatial correspondence and differentiation of female and male happiness

Spatial differentiation between the female and male happiness surfaces was assessed across the same 33,591 grids using grid-level correspondence, contrast structure, and extreme-quartile co-location. Pearson r and Spearman ρ described numerical and rank correspondence. Each continuous outcome was standardized before calculating the female-minus-male contrast, which represents the relative position of the same grid on the two surfaces. Following multivariate spatial-correlation principles (78), we also defined a descriptive symmetric cross-surface Moran statistic (Equation 4):

$$\begin{array}{l} I\_\mathrm{sym}\\ =(n/S_{0})\{\Sigma_{i}{<}_{j}\;w_{\mathrm{ij}}(\begin{array}{l} z\_\mathrm{Mi}\;z\_\mathrm{Fj}+\\ z\_\mathrm{Mj}\;z\_\mathrm{Fi} \end{array})\}/\surd (\Sigma_{i}\;z\_Mi^{2}\;\Sigma_{i}\;z\_Fi^{2}) \end{array}$$ (4)

Significance was evaluated by holding the male surface fixed and globally permuting the female surface 999 times, with two-sided pseudo-*p* values. Global Moran’s I was calculated for the raw and standardized contrasts using the same Rook topology. Local Moran’s I for the standardized contrast used Anselin conditional randomization (79), and the 33,591 local tests were adjusted using the Benjamini-Hochberg procedure (80). Grids with *q* < 0.05 were classified as High-High, Low-Low, High-Low, or Low-High. Extreme high and low sets were defined using their respective 75th and 25th percentiles, and Jaccard indices quantified co-location. Together, these statistics described spatial discordance, local clustering, and extreme co-location between the female and male happiness surfaces.

### 2.6 Ethics statement

This study analysed only publicly accessible, non-private Weibo posts and public profile fields and involved no recruitment, intervention, or direct interaction with users. Usernames, user IDs, URLs, and other direct identifiers were removed before analysis, and all reported results were aggregated to 500 m grids. No raw post text or user-level records are reproduced or redistributed. Data collection used publicly accessible mobile-location pages and did not bypass access controls. The analysis was limited to secondary research use of de-identified data.

## 3 Results

### 3.1 Spatial distribution and bivariate associations of area-level happiness

Figure 3 shows pronounced spatial heterogeneity in female- and male-labelled area-level happiness across Shanghai. Positive expression accounted for 73.64% of the 320,618 female-labelled posts and 74.30% of the 187,089 male-labelled posts. Across the same 33,591 grids, female-labelled scores had a mean of 4.06 (SD = 2.13) and a median of 4.32; male-labelled scores had a mean of 2.94 (SD = 1.71) and a median of 2.87. District-level mean female-labelled happiness was relatively high in Fengxian (6.52), Jinshan (6.94), and Chongming (5.38), whereas mean male-labelled happiness was relatively high in Xuhui (4.88), Putuo (4.53), and Hongkou (3.92). The largest share of female-labelled scores fell within 4 to <5 (26.45%; 8,885 grids), while the largest share of male-labelled scores fell within 2 to <3 (23.27%; 7,816 grids). Negative grid scores accounted for 2.23 and 3.53%, respectively.

**Figure 3:**
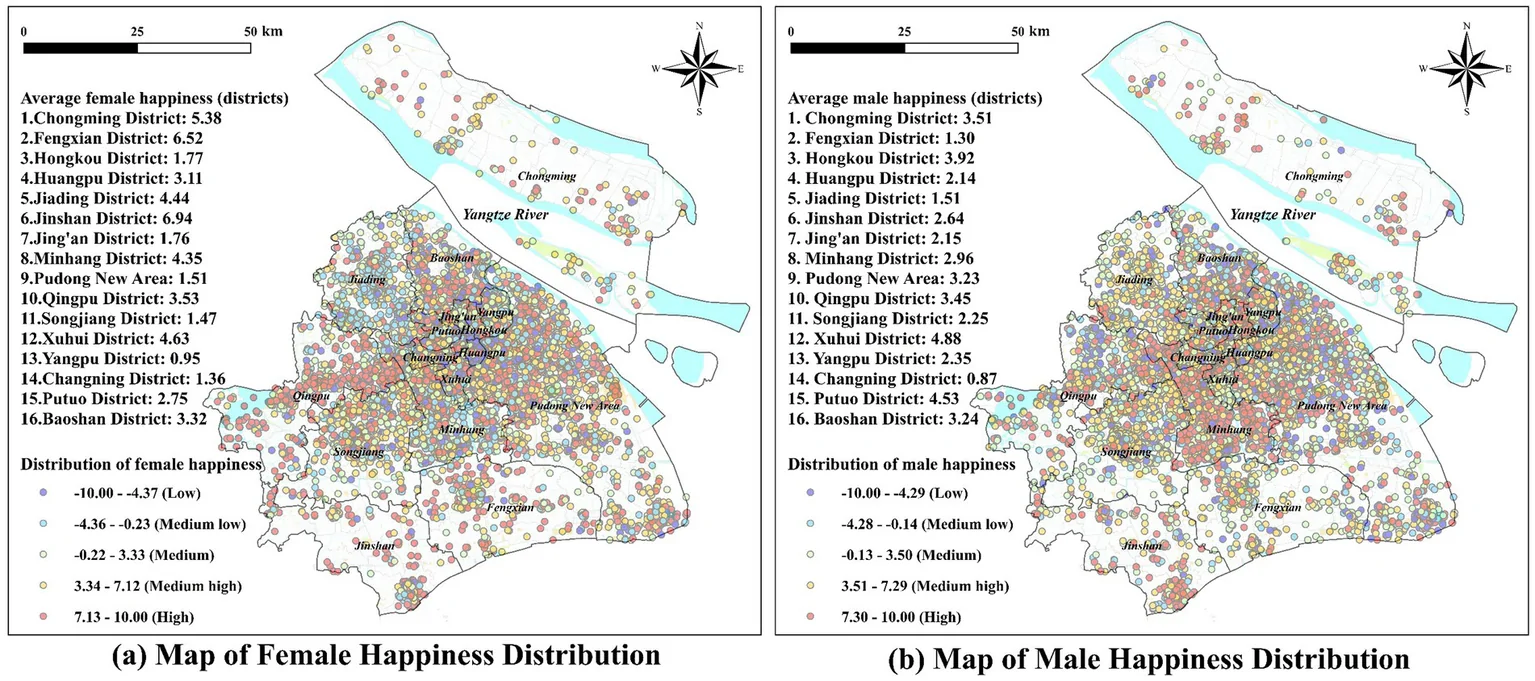
Spatial distribution and district-level mean area-level happiness. **(a)** Female; **(b)** male.

Using the rebuilt binary Rook weights, Global Moran’s I was 0.909 for female-labelled area-level happiness and 0.666 for male-labelled area-level happiness (permutation pseudo-*p* = 0.001 for both; Supplementary Tables S2, S6). Moran’s I for coordinate-grouped out-of-fold residuals remained 0.699 and 0.502 (*p* = 0.001 for both), confirming substantial spatial continuity in both observed outcomes and prediction errors.

Pearson-correlation bubble plots compared unadjusted linear associations between the two area-level happiness outcomes and the built-environment predictors (Figure 4). Most predictors had similar directions for the two platform-label groups, while Population count, the Gini coefficient, and several diversity and accessibility indicators showed clearer contrasts.

**Figure 4:**
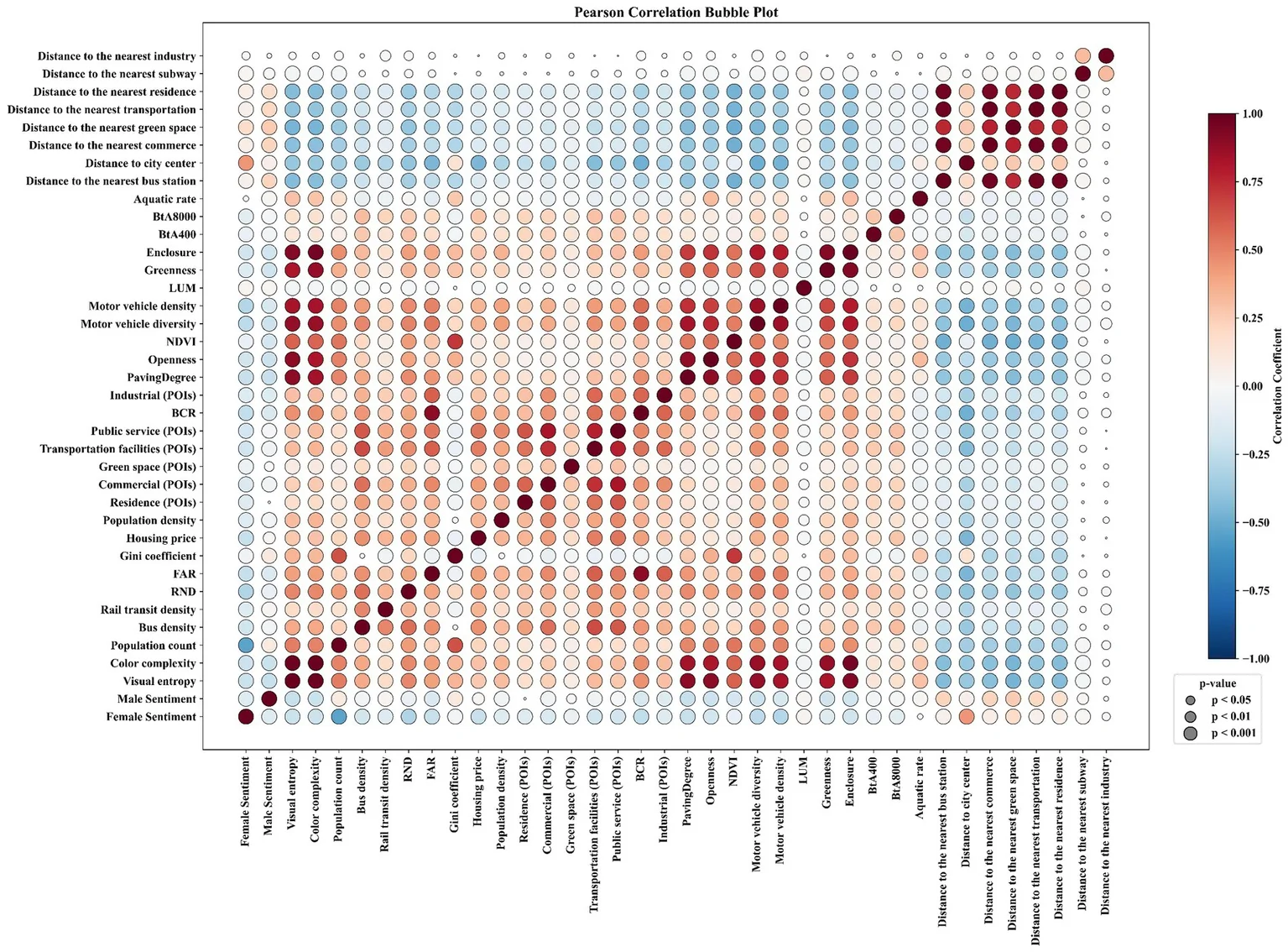
Pearson correlations between happiness and built-environment predictors.

Distance to city center, Distance to the nearest residence, Distance to the nearest commerce, and Distance to the nearest green space were positively correlated with both outcomes, although the association with Distance to city center was stronger for female happiness. Population count was positively correlated with male happiness (r approximately 0.25) and negatively correlated with female happiness (r approximately −0.50). The Gini coefficient showed the same directional contrast (male r approximately 0.25; female r approximately −0.25). Public service, Transportation facilities, Green space, and Commercial POIs had stronger negative correlations with female happiness, whereas most indicators in Design and Distance to Transit showed similar patterns for both groups. These descriptive contrasts identified relationships for subsequent nonlinear model interpretation.

### 3.2 Predictive performance and robustness

On the common 12,187-record labelled holdout, ERNIE-LSTM-TextCNN achieved accuracy = 0.942, macro-F1 = 0.942, and Matthews correlation = 0.885, exceeding Multinomial Naive Bayes (accuracy = 0.883), Bernoulli Naive Bayes (0.925), and the log-odds lexicon (0.893; Supplementary Table S11). Under citywide random five-fold validation, pooled out-of-fold XGBoost performance was *R*^2^ = 0.930 (95% CI, 0.928–0.933), RMSE = 0.451, and MAE = 0.214 for the male-labelled model. The corresponding values for the female-labelled model were *R*^2^ = 0.898 (95% CI, 0.893–0.902), RMSE = 0.681, and MAE = 0.306 (Table 3).

Across validation settings, predictive performance was lower with greater geographic separation. In the primary citywide analysis, coordinate-grouped *R*^2^ was 0.882 for the male-labelled model and 0.759 for the female-labelled model; under the strict spatial-transfer specification defined in Methods, *R*^2^ was 0.371 and 0.356 (Table 3). A separate matched-support benchmark used the same 19,113 grids, 30 predictors, and fold assignments for every model. In this benchmark, fixed-parameter XGBoost achieved random-fold *R*^2^ = 0.568 and 0.587 and buffered spatial *R*^2^ = 0.363 and 0.353 for the male- and female-labelled outcomes, respectively. The corresponding *R*^2^ ranges were 0.315–0.417 and 0.271–0.290 for the male-labelled conventional models, and 0.402–0.449 and 0.310–0.343 for the female-labelled conventional models, under random and buffered validation. XGBoost produced the highest pooled out-of-fold *R*^2^ in all four matched comparisons (Supplementary Tables S12–S14; Supplementary Figure S9).

Calibration followed the same performance gradient. Under random folds, slopes from regressions of observed values on predictions were 1.004 for the male-labelled model and 1.012 for the female-labelled model; intercepts were −0.012 and −0.047, and mean prediction biases were both −0.001. Under coordinate grouping, slopes were 1.006 and 0.952, intercepts were −0.043 and 0.233, and mean biases were +0.025 and −0.038. Under strict spatial transfer, slopes declined to 0.899 and 0.879, intercepts increased to 0.372 and 0.509, and mean biases were approximately +0.013 for both models.

The 95% intervals in Table 3 were estimated from 500 non-spatial row bootstraps of pooled out-of-fold predictions; strict spatial-transfer results are point estimates. Minimum-user sensitivity reduced paired support from 4,832 grids at one user per group to 779 grids at 20 users per group, while pooled random-fold XGBoost *R*^2^ remained positive (male-labelled: 0.718; female-labelled: 0.842; Supplementary Table S10; Supplementary Figure S8). The streetscape-support analysis identified 14,478 grids with zero values across all eight streetscape indicators. On the same 19,113 supported grids, removing the streetscape predictors reduced random-fold *R*^2^ from 0.568 to 0.484 for the male-labelled outcome and from 0.587 to 0.523 for the female-labelled outcome; full random and buffered results are reported in Supplementary Table S13 and Supplementary Figure S9.

### 3.3 Global and local built-environment associations

#### 3.3.1 Global importance and predictor-level nonlinear patterns

Random-fold out-of-fold SHAP separated broader contextual predictors from local built-environment variation (Figures 5, 6). In the male model, Population count ranked first (mean absolute SHAP = 0.647; 21.4% of total absolute attribution), followed by Greenness (0.621; 20.5%), the Gini coefficient (0.318; 10.5%), Distance to city center (0.244; 8.1%), and NDVI (0.171; 5.6%). In the female model, NDVI ranked first (0.673; 21.2%), followed by the Gini coefficient (0.564; 17.8%), Population count (0.411; 13.0%), Greenness (0.318; 10.0%), and Distance to city center (0.307; 9.7%).

**Figure 5:**
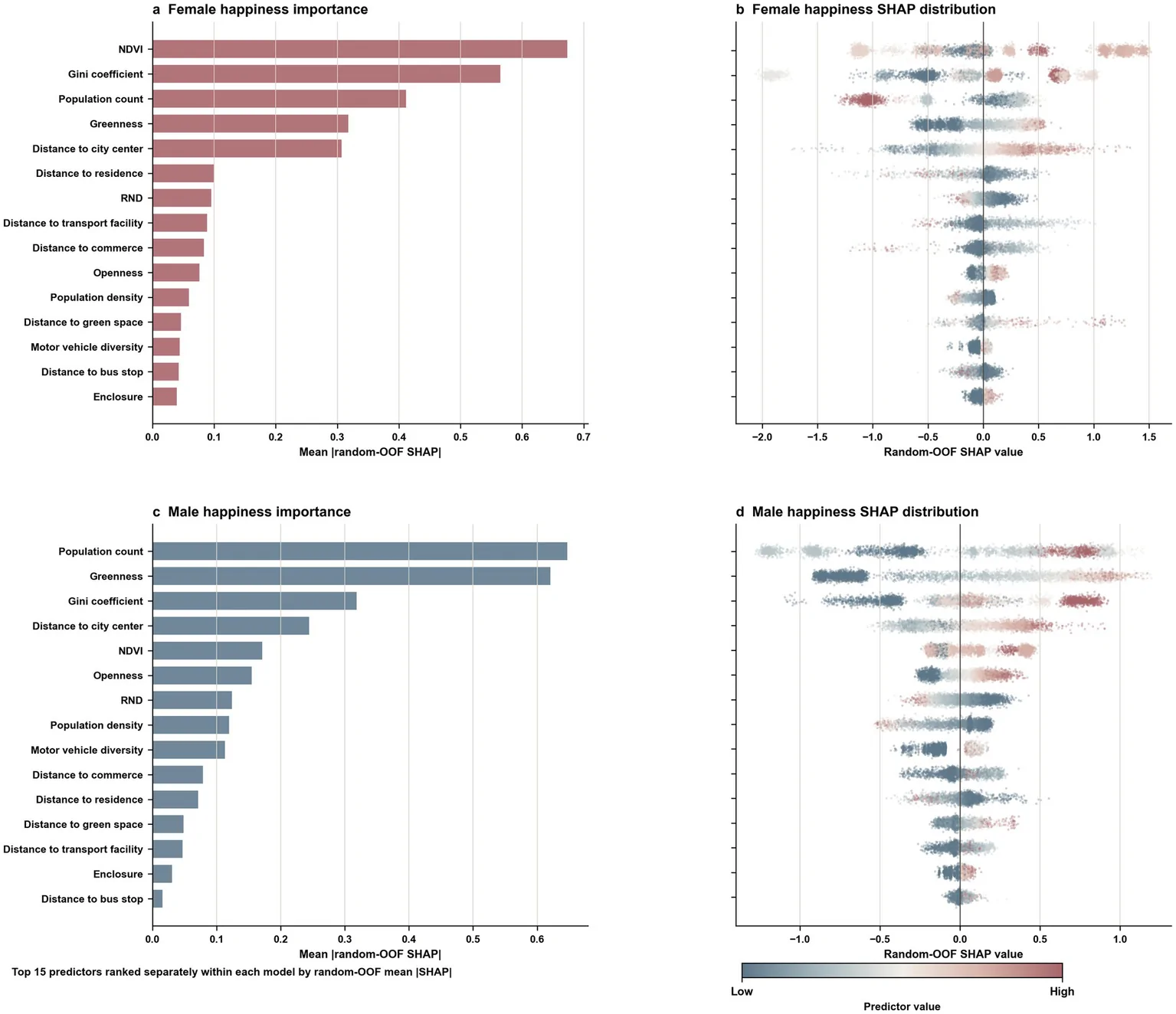
Out-of-fold SHAP importance and distributions. **(a)** Female-labelled happiness importance; **(b)** female-labelled happiness SHAP distribution; **(c)** male-labelled happiness importance; **(d)** male-labelled happiness SHAP distribution. The panels compare the 15 leading predictors retained in the paired PDP–SVC analysis of the female- and male-labelled happiness models.

**Figure 6:**
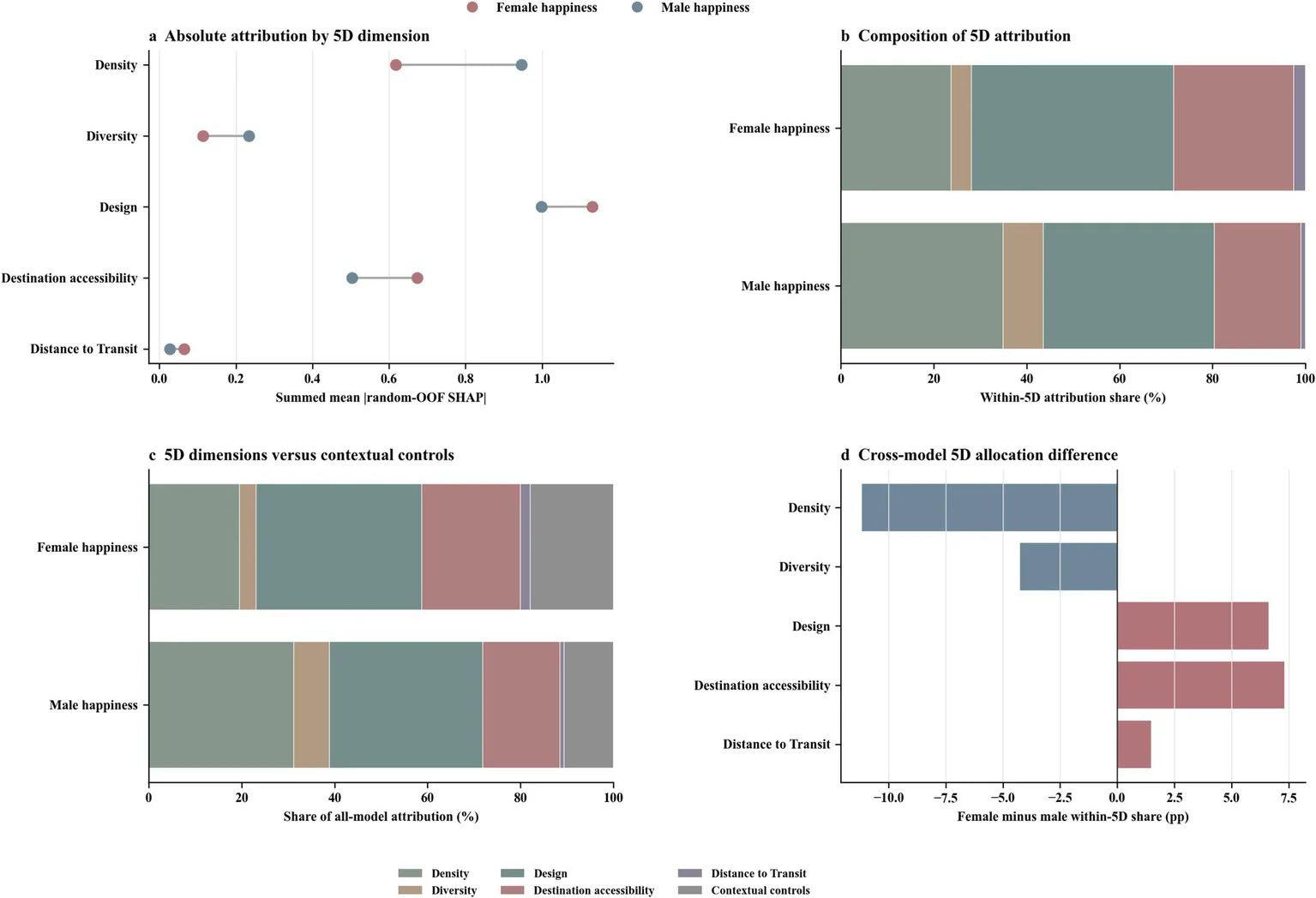
SHAP contributions under the 5D classification. **(a)** Absolute 5D attribution; **(b)** within-5D composition; **(c)** attribution shares of the 5D dimensions and socioeconomic variables; **(d)** female-minus-male difference in attribution share.

Under the 5D classification, Design contributed the largest share in both models, but its internal composition differed. In the female model, Design accounted for 43.5% of 5D absolute attribution, Destination accessibility for 25.9%, and Density for 23.8%. In the male model, Design accounted for 36.9%, Density for 34.9%, and Destination accessibility for 18.6%. The Gini coefficient and Housing price together accounted for 17.9% of total absolute attribution in the female model and 10.6% in the male model.

Figures 7–11 pair PDP and SVC results for each retained predictor within the five 5D dimensions. PDPs show centred full-sample curves with 2.5th–97.5th percentile ranges across five random-fold training models, while the maps show female-minus-male differences in absolute out-of-fold SVC. Predictors excluded from the strict local analysis by support or fold-specific collinearity screening are shown only in the corresponding sensitivity panels.

**Figure 7:**
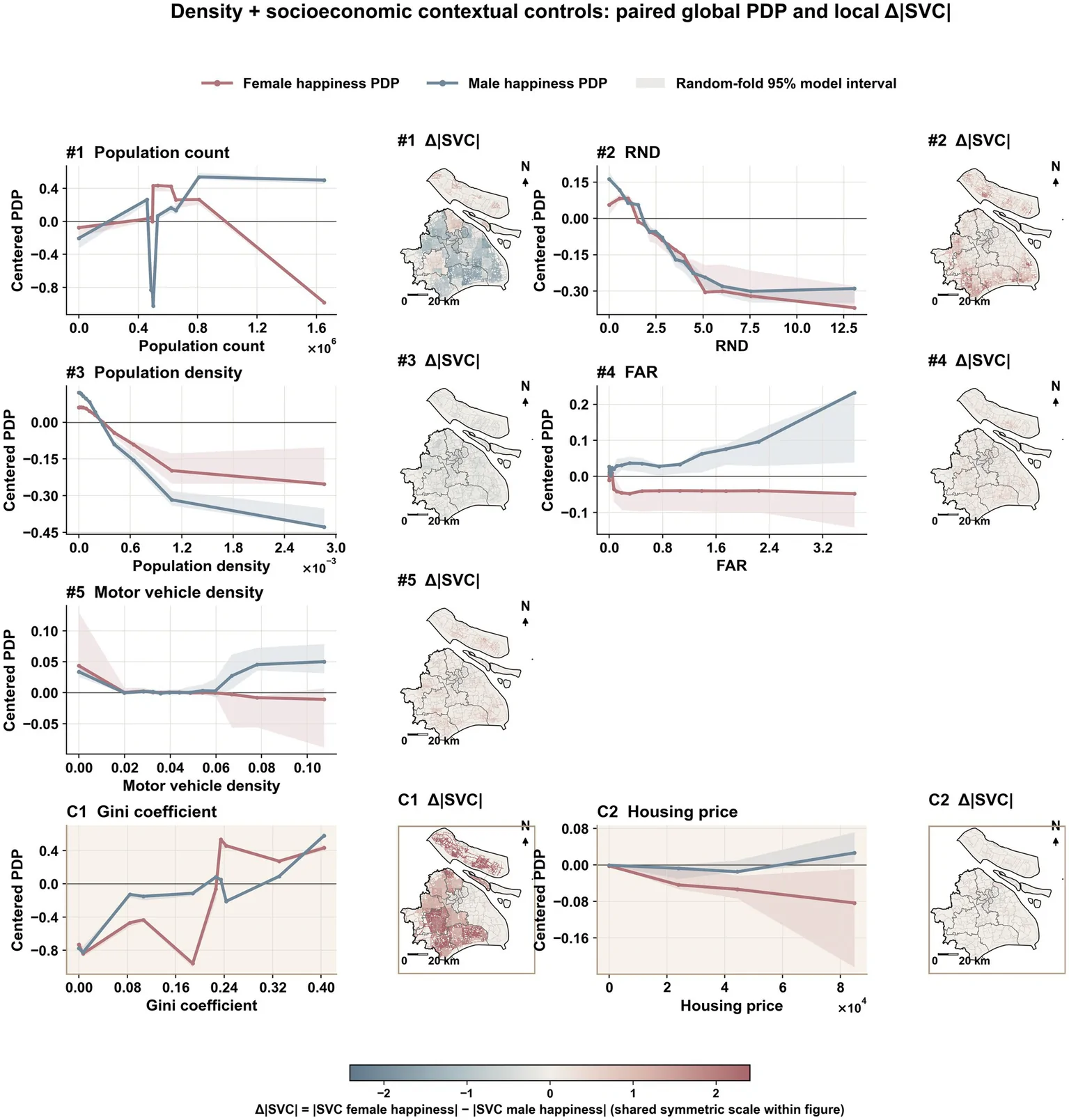
Density and socioeconomic variables: global nonlinearity and local spatial contributions. Paired centred PDPs (left) and female-minus-male differences in absolute SVC (right) are shown for retained predictors. Solid curves show the full-sample PDP, and shading shows the 2.5th–97.5th percentile range across five random-fold training models. Maps show the Shanghai municipal, district, and township boundaries.

**Figure 8:**
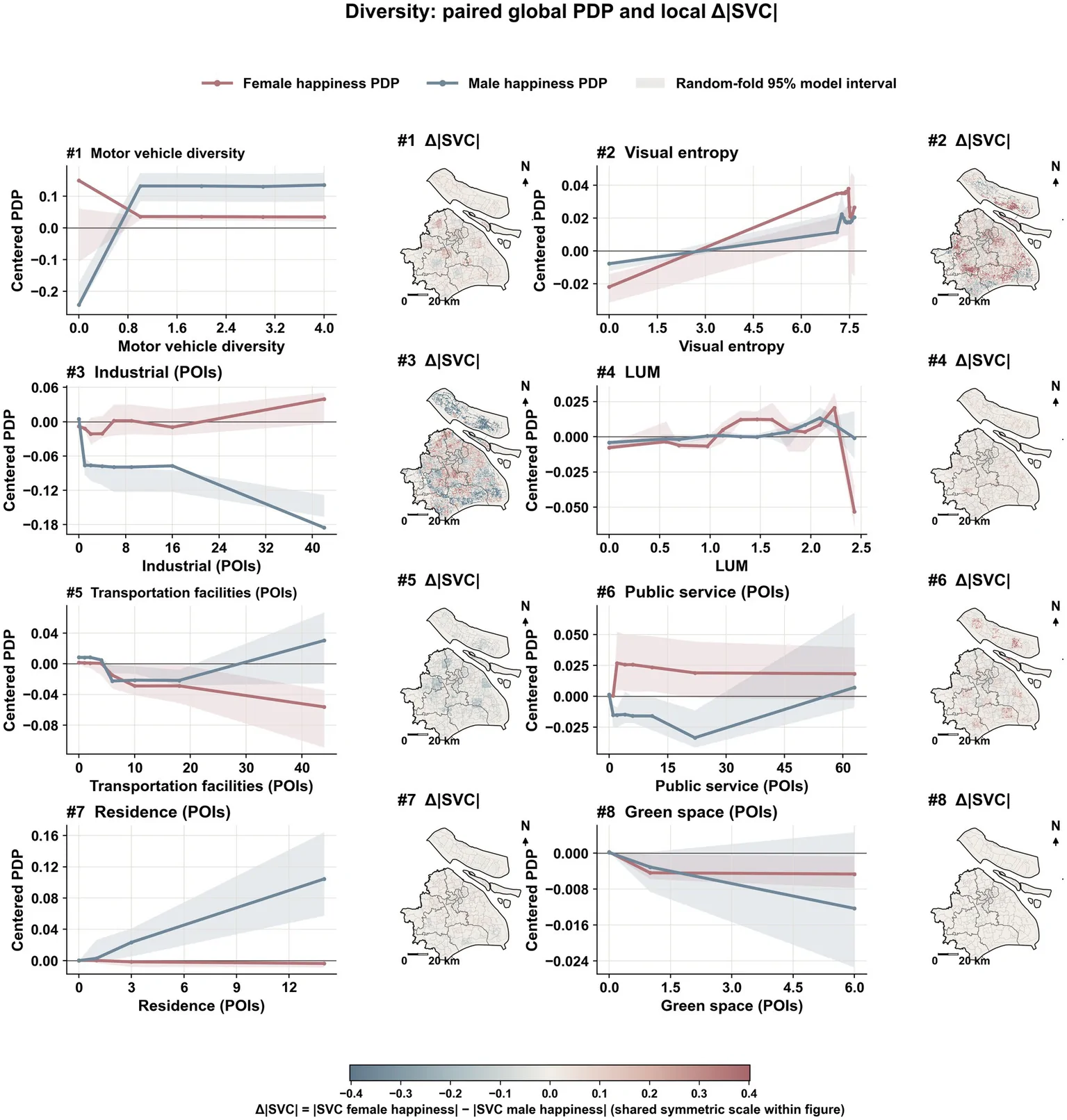
Diversity: global nonlinearity and local spatial contributions. Paired centred PDPs (left) and female-minus-male differences in absolute SVC (right) are shown for retained predictors. Solid curves show the full-sample PDP, and shading shows the 2.5th–97.5th percentile range across five random-fold training models. Maps show the Shanghai municipal, district, and township boundaries.

**Figure 9:**
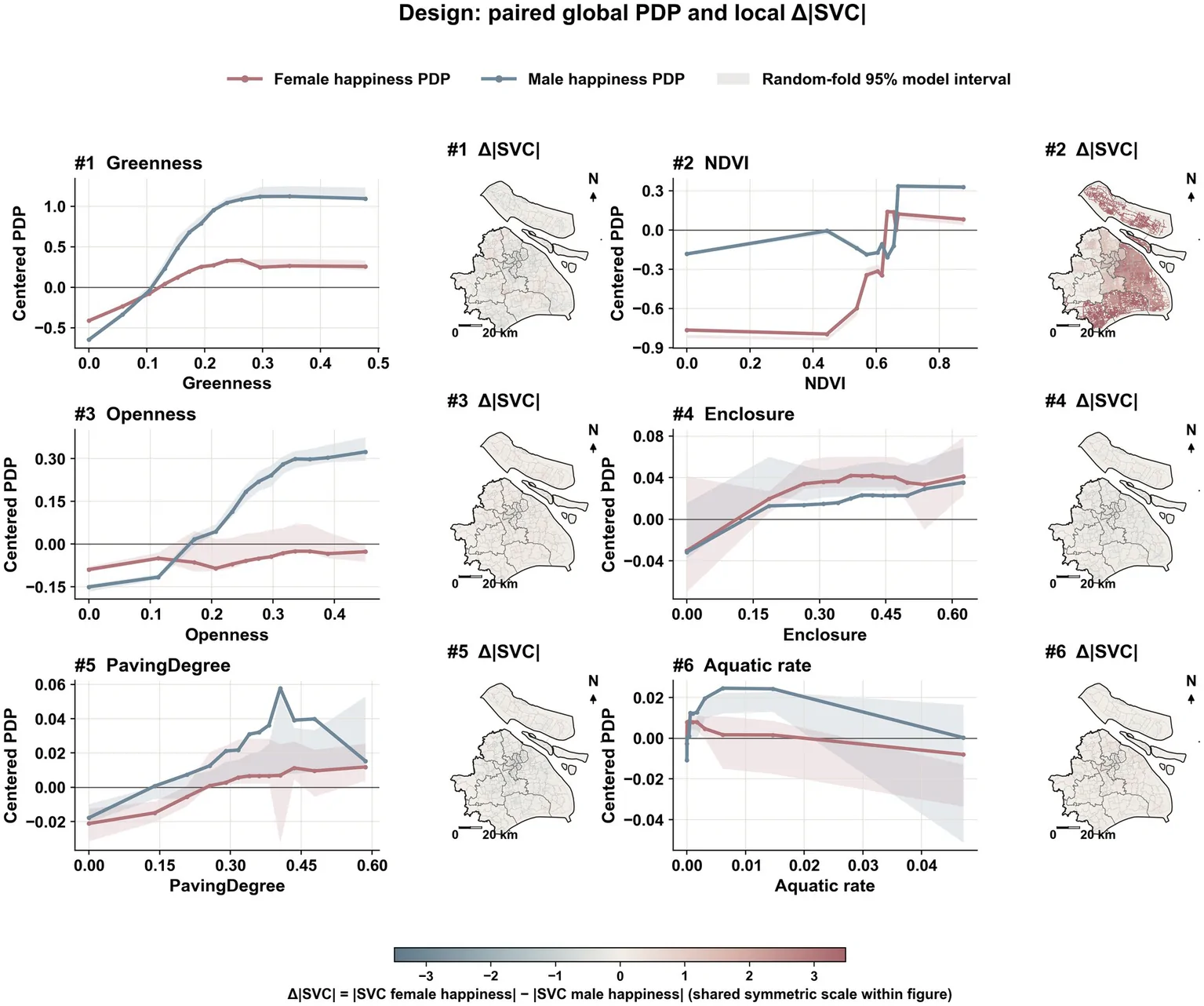
Design: global nonlinearity and local spatial contributions. Paired centred PDPs (left) and female-minus-male differences in absolute SVC (right) are shown for retained predictors. Solid curves show the full-sample PDP, and shading shows the 2.5th–97.5th percentile range across five random-fold training models. Maps show the Shanghai municipal, district, and township boundaries.

**Figure 10:**
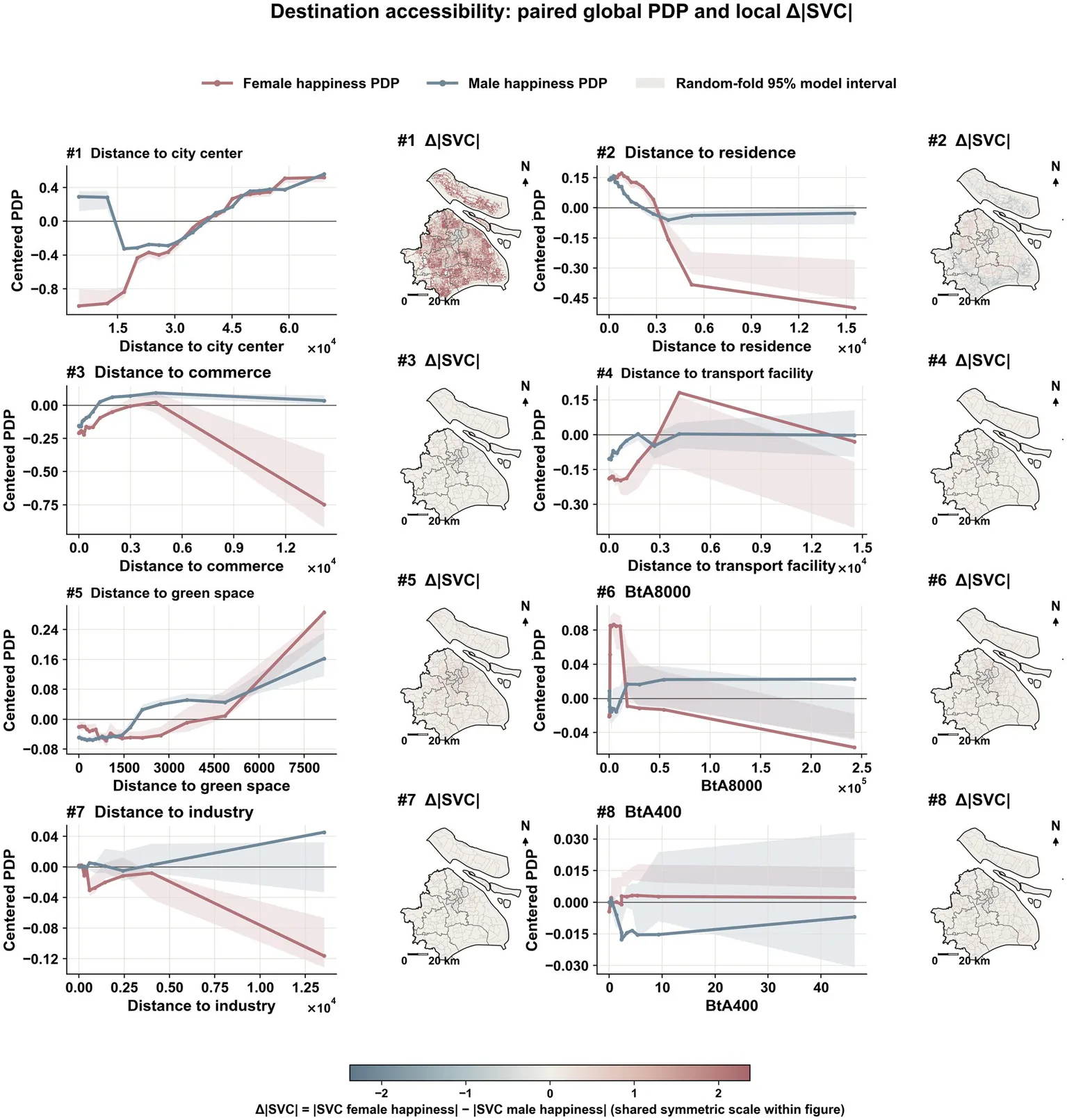
Destination accessibility: global nonlinearity and local spatial contributions. Paired centred PDPs (left) and female-minus-male differences in absolute SVC (right) are shown for retained predictors. Solid curves show the full-sample PDP, and shading shows the 2.5th–97.5th percentile range across five random-fold training models. Maps show the Shanghai municipal, district, and township boundaries.

**Figure 11:**
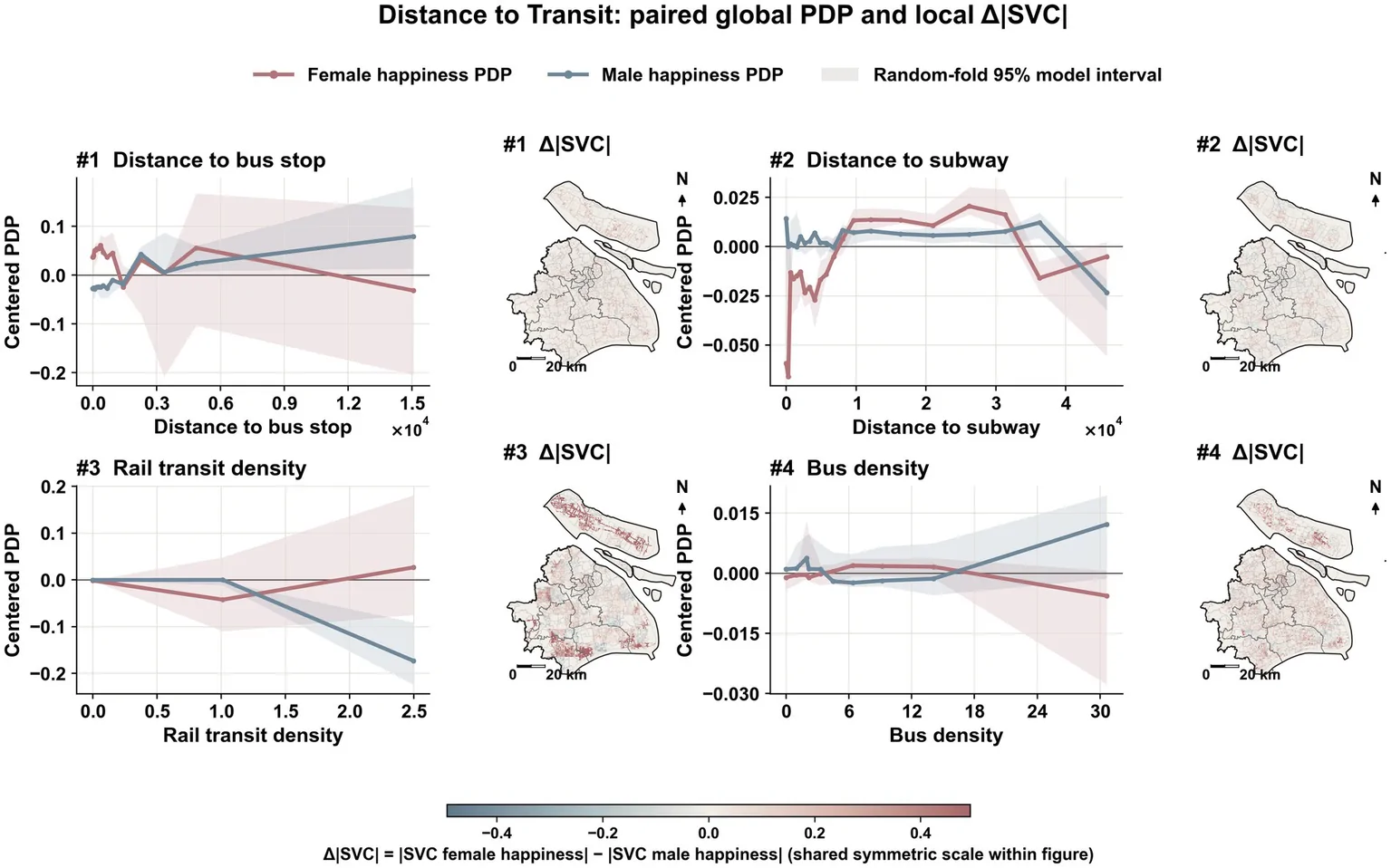
Distance to transit: global nonlinearity and local spatial contributions. Paired centred PDPs (left) and female-minus-male differences in absolute SVC (right) are shown for retained predictors. Solid curves show the full-sample PDP, and shading shows the 2.5th–97.5th percentile range across five random-fold training models. Maps show the Shanghai municipal, district, and township boundaries.

Within Density, Population count produced the largest PDP contrast. In the extended sensitivity model, mean absolute SVC was 0.685 for the female model and 1.321 for the male model. The median absolute-SVC difference was −0.780, and 20.7% of grids had a larger female-model contribution. The 17 area-level Population count values capture regional population context, and their distribution varied across spatial folds. In the main model, both RND curves decreased with increasing values, with the female curve lower at the upper end. The median absolute-SVC difference was +0.285, and the female model had the larger contribution in 83.5% of grids. Population density showed the opposite pattern: the median difference was −0.235, and only 6.9% of grids favoured the female model. Median differences for FAR and Motor vehicle density were +0.081 and +0.135. The extended Gini-coefficient sensitivity model strongly favoured the female model (mean absolute SVC, 1.386 versus 0.126; median difference, +1.319), again at area-level support. For Housing price, mean absolute SVC was 0.075 in the female model and 0.018 in the male model, with differences concentrated in a limited spatial subset (Figure 7).

Differences within Diversity were concentrated in Industrial (POIs), Visual entropy, and Transportation facilities (POIs). As Industrial (POIs) increased, the female PDP shifted from near zero to slightly positive, whereas the male curve continued to decline. Its median absolute-SVC difference was −0.029, but district medians ranged from −0.258 in Chongming to +0.113 in Yangpu. Both Visual entropy curves increased, with the female curve higher at larger values. The median difference was +0.041, and 64.1% of grids favoured the female model. The female PDP for Transportation facilities (POIs) declined, whereas the male curve turned upward at higher values; its median local contribution difference was −0.027. After adaptive training-fold support was applied, mean absolute SVC for Green space (POIs) was 0.0037 in the female model and 0.0020 in the male model. The dimension-level pattern remained dominated by Industrial (POIs) and Visual entropy (Figure 8).

Within Design, both Greenness PDPs changed from negative to positive at approximately 0.1, but the male curve increased more sharply. The median absolute-SVC difference was −0.171, and only 28.8% of grids favoured the female model. Enclosure and PavingDegree also favoured the male model, with median differences of −0.122 and −0.117. The male Openness PDP became positive near 0.15 and continued to rise, while the female model had the larger absolute local contribution in 78.5% of grids. Aquatic rate also favoured the female model (median difference, +0.063; 65.7% of grids). The extended sensitivity model provided a regional-scale map for NDVI. Mean absolute SVC was 2.162 in the female model and 0.143 in the male model, with a median difference of +2.484. NDVI was represented by 17 area-level values, which capture vegetation context at a broader spatial support than the 500 m grid (Figure 9).

Distance to city center dominated the differences within Destination accessibility. Both PDPs followed an initial decline followed by an increase. The female curve was lower near the centre and gradually converged as distance increased. The median absolute-SVC difference reached +1.451, and the female model had the larger contribution in 94.1% of grids. District medians were +1.896 in Jinshan and +1.769 in Chongming, compared with −0.449 in Hongkou. The female PDP for Distance to residence fell sharply beyond approximately 3 km, although its median local contribution difference was −0.023, indicating a slightly larger male-model contribution in more than half of the grids. At higher values of Distance to green space, both PDPs increased. Its median absolute-SVC difference was +0.111, and 84.6% of grids favoured the female model (Figure 10).

Within Distance to Transit, PDP contrasts were most pronounced in the distribution tails. At high values of Distance to bus stop, the female curve changed from positive to negative while the male curve continued to increase. Rail transit density and Bus density also showed opposing directions at their upper ranges, although uncertainty across random-fold models widened. In the strict spatial sample, median absolute-SVC differences for Bus density, Distance to subway, and Distance to bus stop were +0.046, +0.011, and +0.021. The female model had the larger contribution in 71.1, 58.7, and 68.5% of grids. With adaptive training-fold support, mean absolute SVC for Rail transit density was 0.145 in the female model and 0.042 in the male model. Its median difference was +0.029, and 59.7% of grids favoured the female model. The combined PDP and SVC results show that gender differences within this dimension varied by indicator, value range, and location (Figure 11).

#### 3.3.2 Spatial contribution patterns across grids and districts

The female and male models shared the same dominant 5D dimension in 43.3% of grids (Figure 12). Design formed the largest common component. Density dominated a wider area in the male model, whereas Destination accessibility formed clearer female-model clusters. The transition matrix showed that some grids dominated by Design or Destination accessibility in the female model shifted to Design or Density in the male model. The two models therefore relied on different environmental information at many of the same locations.

**Figure 12:**
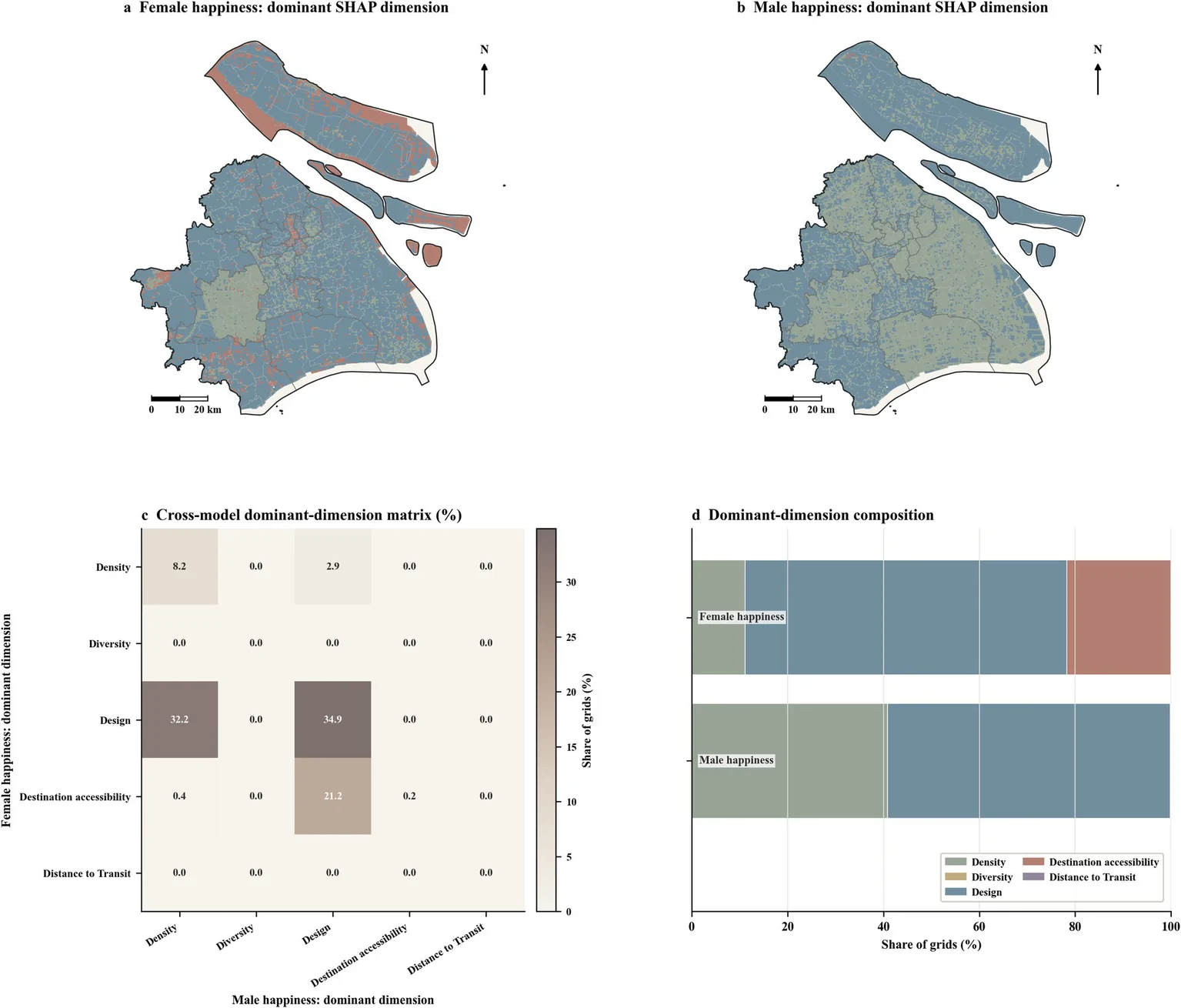
Spatial structure of dominant 5D dimensions. **(a,b)** Dominant dimension in the female and male happiness models; **(c)** transition matrix; **(d)** dimensional composition.

Table 4 reports female- and male-model ranks, mean absolute SHAP values, and shares of total absolute SHAP for the ten leading predictors in the two 36-variable random-fold analyses.

**Table 4:** Leading out-of-fold SHAP predictors in female and male happiness models.

Predictor	Female rank	Female mean absolute SHAP	Female share	Male rank	Male mean absolute SHAP	Male share
Population count	3	0.4112	13.0%	1	0.6467	21.4%
NDVI	1	0.6730	21.2%	5	0.1710	5.6%
Gini coefficient	2	0.5643	17.8%	3	0.3184	10.5%
Greenness	4	0.3176	10.0%	2	0.6206	20.5%
Distance to city center	5	0.3071	9.7%	4	0.2441	8.1%
Openness	10	0.0761	2.4%	6	0.1547	5.1%
Distance to the nearest residence	6	0.0998	3.1%	—	—	—
RND	7	0.0955	3.0%	7	0.1239	4.1%
Population density	—	—	—	8	0.1192	3.9%
Distance to the nearest transportation	8	0.0885	2.8%	—	—	—
Distance to the nearest commerce	9	0.0832	2.6%	10	0.0785	2.6%
Motor vehicle diversity	—	—	—	9	0.1129	3.7%

Figure 13 summarizes strict spatial out-of-fold SVCs for all 29 retained 5D predictors using observed local contrasts, within-dimension rankings, and district-level median differences. BCR, Commercial (POIs), and Color complexity were excluded by fold-specific variance inflation factor screening. Regional-scale sensitivity maps for Population count and NDVI are presented in Figure 7. Based on combined mean absolute SVC, RND and Population density ranked first within Density; Industrial (POIs) and Visual entropy within Diversity; Greenness and Enclosure within Design; Distance to city center and Distance to residence within Destination accessibility; and Bus density and Rail transit density within Distance to Transit. Housing price is reported separately as a socioeconomic contextual variable in Figure 7.

**Figure 13:**
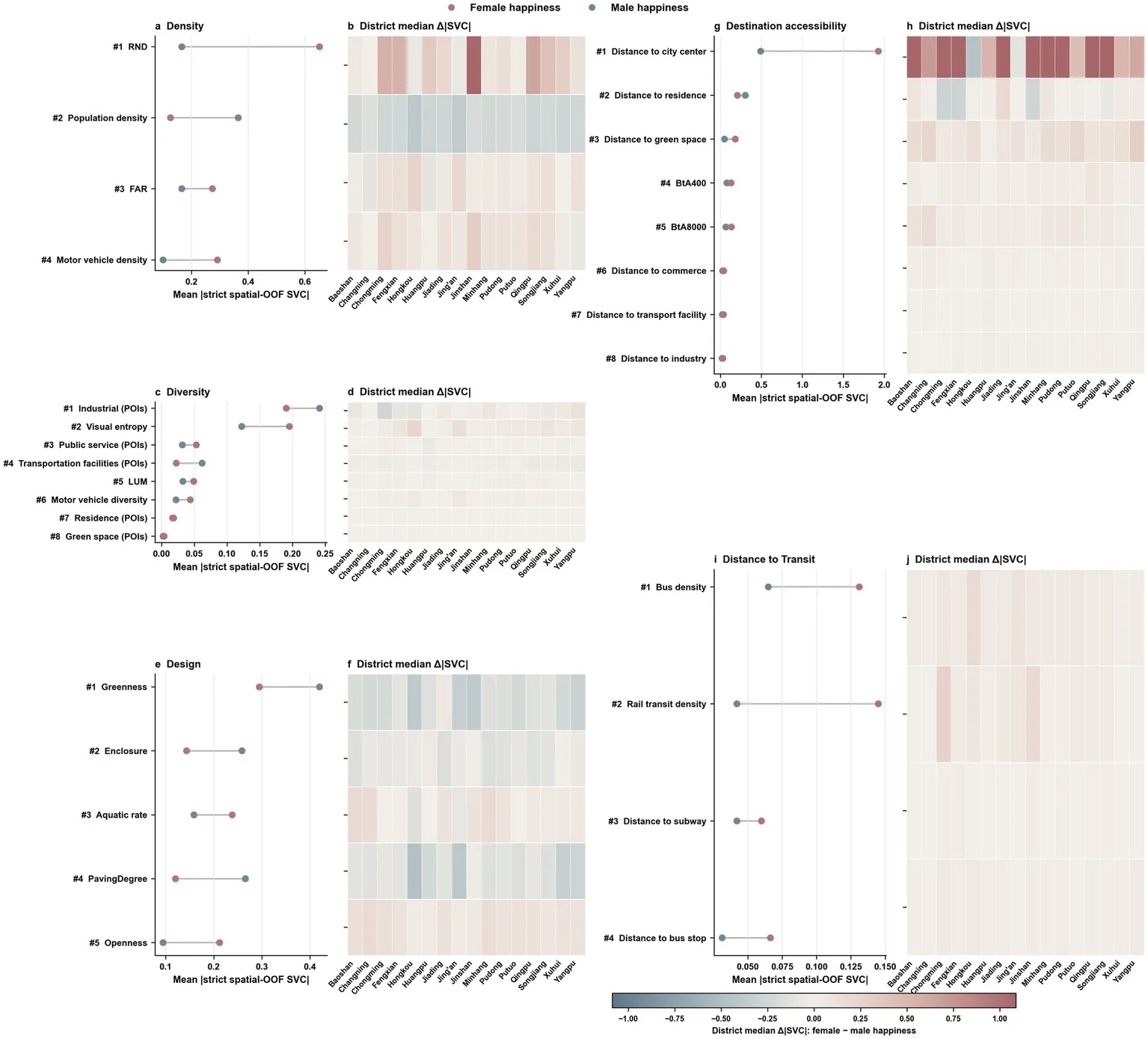
Predictor rankings and district differences in spatial contributions. **(a,c,e,g,i)** Mean absolute SVC for the female and male models; **(b,d,f,h,j)** district medians of the female-minus-male absolute-SVC difference.

Across the five paired dimension-level contrasts, Destination accessibility had the largest median female-minus-male difference in mean absolute SVC (+0.205; Q1 = +0.124; Q3 = +0.280), and 95.1% of grids favoured the female model. Design had a negative median difference (−0.041), and only 32.0% of grids favoured the female model. Distance to Transit had a median difference of +0.040 (Q1 = +0.011; Q3 = +0.082), with larger female-model contributions in 84.0% of grids. Moran’s I for the five dimension-level differences ranged from 0.571 to 0.788 (permutation pseudo-*p* = 0.001 for all), demonstrating that the direction and spatial extent of contribution differences varied among dimensions.

District medians showed that gender differences within each dimension arose from different indicators and locations. Within Density, RND favoured the female model most strongly in Jinshan and Qingpu, whereas Population density had a negative median difference in all 16 districts. Within Diversity, Visual entropy favoured the female model in Hongkou and Jing’an, while Industrial (POIs) ranged from −0.258 in Chongming to +0.113 in Yangpu. Within Design, Greenness favoured the male model most strongly in Hongkou and Jinshan, whereas Aquatic rate favoured the female model in Baoshan and Minhang. The principal spatial gradient in Destination accessibility came from Distance to city center. Within Distance to Transit, Bus density had a positive district median in all 16 districts, reaching +0.172 in Hongkou and +0.108 in Jing’an.

### 3.4 Gendered spatial differentiation in area-level happiness

Female-labelled area-level happiness exceeded male-labelled area-level happiness by 1.115 points on average and 1.060 points at the median. Across matched grids, Pearson r was 0.325 and Spearman ρ was 0.273. The quartile-flow analysis placed 30.9% of grids in the same quartile, 35.2% in adjacent quartiles, and 6.03% in opposite extreme quartiles (Figure 14a). The symmetric cross-surface Moran statistic was 0.171 (permutation pseudo-*p* = 0.001). Random-fold out-of-fold mean absolute SHAP values for all 36 predictors were then summarized by the 5D dimensions and contextual variables. Design contributed the largest share in both models; Destination accessibility was more prominent in the female-labelled model, whereas Density was more prominent in the male-labelled model (Figure 14b).

**Figure 14:**
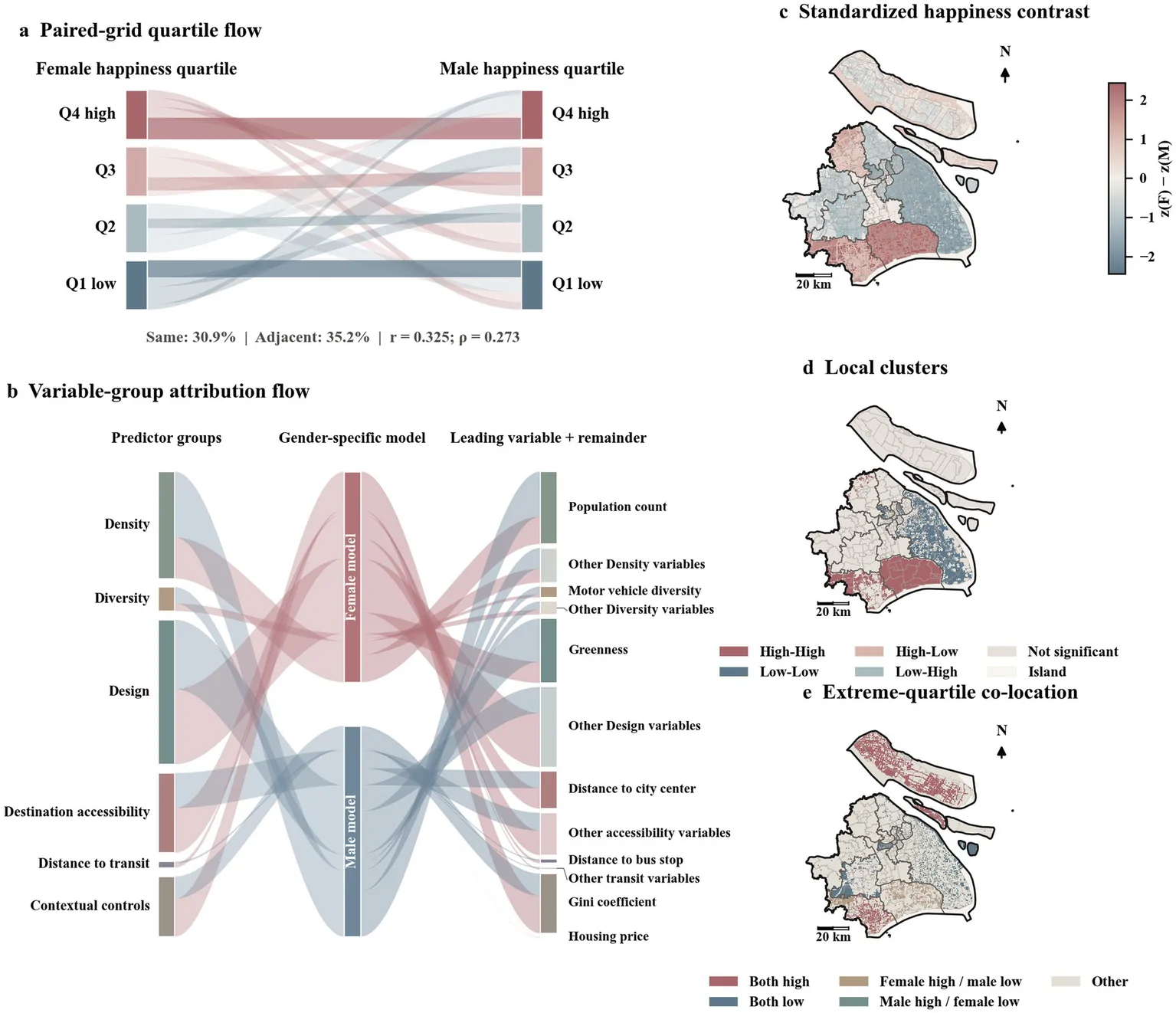
Gendered spatial correspondence, model attribution, and local differentiation. **(a)** Paired-grid quartile-flow Sankey diagram; **(b)** random-fold out-of-fold mean absolute SHAP attribution flow for all 36 predictors, summarized by the 5D dimensions and contextual controls; **(c)** standardized happiness contrast clipped to the municipal boundary; **(d)** local clusters of the standardized contrast; **(e)** extreme-quartile co-location. Ribbon width represents within-model attribution share. Maps show the Shanghai municipal, district, and township boundaries.

Both the raw female-minus-male difference and the standardized contrast were strongly clustered, with Moran’s I values of 0.948 and 0.913 (permutation pseudo-*p* = 0.001 for both; Figure 14c). After conditional randomization and multiple-testing correction, 4,137 grids formed High-High clusters and 3,636 formed Low-Low clusters. One High-Low grid and one Low-High grid also remained significant at *q* < 0.05 (Figure 14d). Most relative differences therefore formed contiguous same-direction regions, with only two significant local outliers.

Extreme-quartile co-location provided a complementary view. Joint high and joint low areas accounted for 10.90 and 8.62% of all grids, with Jaccard indices of 0.279 and 0.208. Female-high/male-low grids accounted for 3.67%, while male-high/female-low grids accounted for 2.36% (Figure 14e). These combinations identify localized divergence within a partly shared citywide structure.

Grid-level pairing across the five 5D dimensions linked the happiness contrasts to environmental attribution (Table 5 and Figure 15). Destination accessibility and Distance to Transit generally contributed more to the female-labelled model, whereas Design more often favoured the male-labelled model. The corresponding female-minus-male SVC differences formed spatially coherent clusters across all five dimensions.

**Table 5:** Paired 5D spatial contribution intensity in female and male happiness models.

5D dimension	Female median mean absolute SVC	Male median mean absolute SVC	Median difference [Q1, Q3]	Grids with larger female contribution	Moran’s I
Density	0.278	0.191	+0.086 [+0.001, +0.238]	75.4%	0.788
Diversity	0.067	0.058	+0.009 [−0.018, +0.032]	58.5%	0.571
Design	0.195	0.237	−0.041 [−0.096, +0.018]	32.0%	0.607
Destination accessibility	0.340	0.122	+0.205 [+0.124, +0.280]	95.1%	0.632
Distance to Transit	0.082	0.039	+0.040 [+0.011, +0.082]	84.0%	0.653

**Figure 15:**
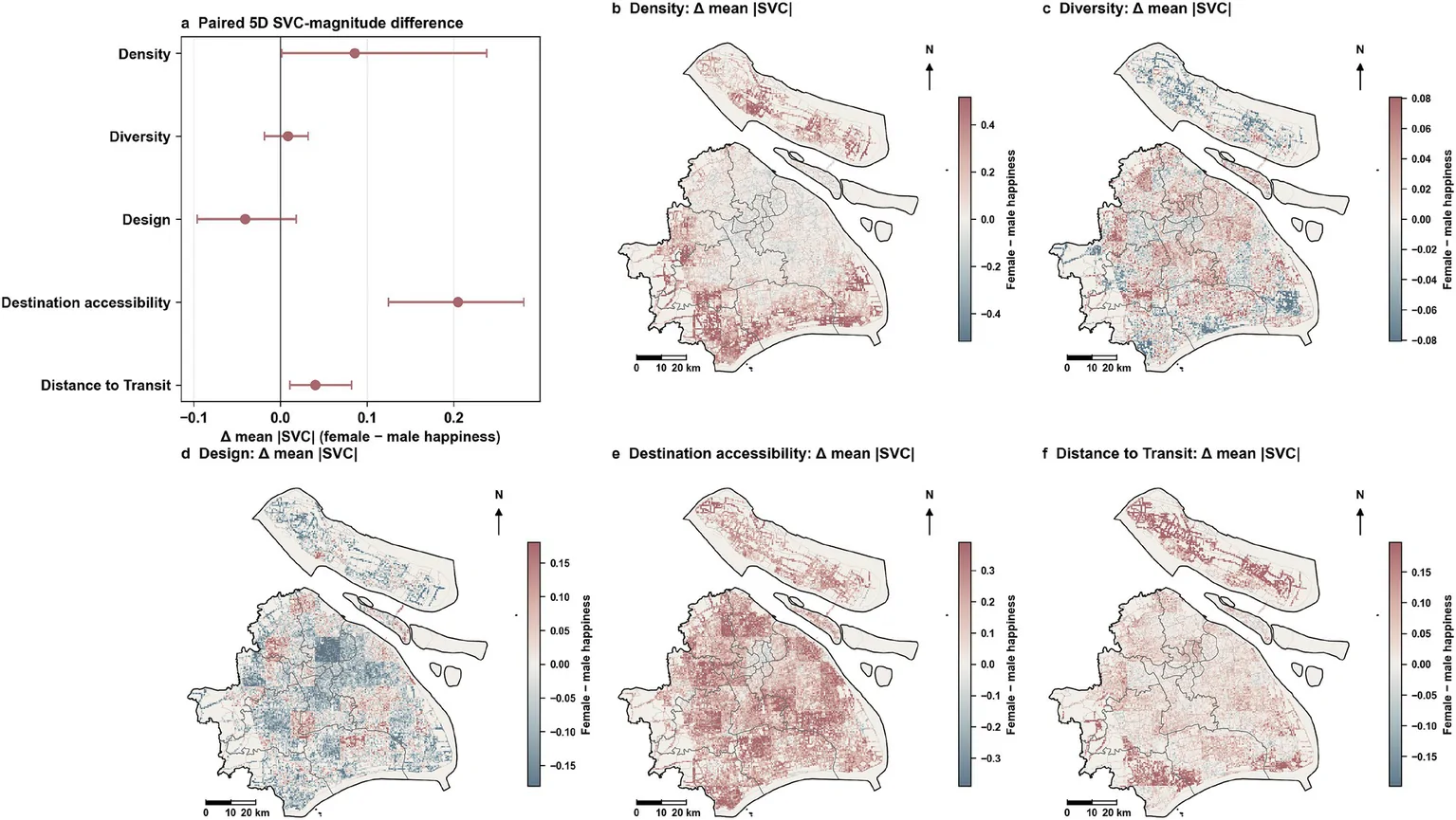
Gender differences in spatial contribution intensity across 5D dimensions. **(a)** Paired dimension-level differences; **(b-f)** spatial distributions of the five dimensions.

## 4 Discussion

### 4.1 Gendered spatial differentiation in area-level happiness

Female- and male-labelled area-level happiness differed in both spatial distribution and local built-environment contribution. Grid-level correspondence was modest (*r* = 0.325; *ρ* = 0.273), the symmetric cross-surface Moran statistic was 0.171, and Moran’s I for the standardized female-minus-male contrast reached 0.913. After multiple-testing correction, 4,137 High-High and 3,636 Low-Low grids together accounted for 23.1% of the 33,591 analytical grids, while 6.03% occupied opposite extreme quartiles. These contiguous differences extend research on residential distributions, potential accessibility, activity spaces, and place co-presence (50–52) to the geography of area-level happiness. Gendered perceptions of urban space can shape entry, lingering, and avoidance (81); the Shanghai results show where those broader processes coincide with persistent differences in area-level affective expression. Niu et al. (41) also identified gendered and spatially heterogeneous patterns in urban emotion derived from geotagged social media; the present paired-grid analysis further locates where gender differences in happiness and environmental contribution coincide.

The environmental composition of the two happiness surfaces also differed. Female- and male-labelled models shared the same dominant 5D dimension in only 43.3% of grids. Destination accessibility and Distance to Transit contributed more to the female-labelled model in 95.1 and 84.0% of grids, whereas Design contributed more to the male-labelled model in 68.0%. Accessibility and transit distance represent access to opportunities, mobility burdens, and time constraints associated with care (82, 83). Mobile-phone, ride-hailing, and GPS studies have similarly documented gender differences in travel distance, activity space, and time spent within neighbourhoods (84–86). Perceived safety and the fit between individual needs and environmental conditions further shape affective experience and environmental avoidance (87–90). These mechanisms provide a plausible context for the different environmental associations observed between the two platform-label groups within the same grids. Sun et al. (91) reported stronger associations of green-space accessibility with men’s life satisfaction and of visible greenery with women’s life satisfaction. In Shanghai, NDVI led the female SHAP ranking, whereas street-view Greenness showed larger male-model contributions in most grids. This contrast suggests that gender patterns vary with the exposure measure and outcome. Gui et al. (92) reported stronger associations of accessibility and residential density with men’s subjective wellbeing. Here, Destination accessibility and Distance to Transit were more prominent in the female-labelled model, possibly because the two studies differ in outcome definition and environmental measurement.

The combined maps distinguish planning-relevant area types. Areas with large differences in both area-level happiness and environmental contribution provide the clearest evidence of gendered spatial differentiation. Areas with similar scores but divergent contributions reveal different environmental pathways to comparable outcomes, while areas with divergent scores but similar contributions point to social or activity-pattern factors beyond the measured environment. Activity-space and destination exposure represent everyday experience beyond residence, and co-presence captures shared location without direct interaction (93). This interpretation turns the cross-surface results into a focused agenda for field observation, resident surveys, mobility diaries, and local safety audits.

### 4.2 Geographic validation and multiscale environmental attribution

Geographic separation reduced predictive performance even after sample support and predictors were held constant. On the same 19,113 grids and 30 predictors, XGBoost *R*^2^ changed from 0.587 to 0.353 for the female-labelled outcome and from 0.568 to 0.363 for the male-labelled outcome between random and buffered spatial folds. Ridge regression, PLS regression, KNN, and Linear SVR all produced lower point-estimate *R*^2^ values under both validation schemes. The transfer penalty across these model families indicates that geographic separation constrained prediction in new areas. Relationships between environment and affect vary with location, land use, time, and observational scale (94, 95); the same-support design identifies the spatial component of this loss. Duan et al. (96) similarly used Weibo and machine learning to relate urban youth sentiment to Shanghai’s built environment. The present same-support results add gender-labelled outcomes and show that prediction deteriorates across geographically separated areas even when sample support and predictors are held constant.

The SHAP results separated regional context from local built-environment variation. NDVI ranked first in the female-labelled model (mean absolute SHAP = 0.673; 21.2%), followed by the Gini coefficient and Population count. Population count ranked first in the male-labelled model (0.647; 21.4%), followed by Greenness (0.621; 20.5%) and the Gini coefficient. Under the 5D classification, Design, Destination accessibility, and Density accounted for 43.5, 25.9, and 23.8% of female-model 5D attribution; Design and Density accounted for 36.9 and 34.9% in the male model. Functional mix has a context-dependent relationship with urban vitality (55, 56), and tree-based models with SHAP have been used to identify streetscape contributions (97). The observed-support classification clarifies which patterns reflect regional context and which vary at the 500 m grid scale. A study of tourists in traditional villages in Fuzhou likewise ranked Openness, Greenness, and Color complexity as the strongest environmental predictors and reported nonlinear favourable ranges (24). Shanghai partly shares this visual-environment pattern, but its leading attributions also involved Population count, NDVI, the Gini coefficient, and Distance to city center, and their rankings differed between platform-gender groups. Compared with the Fuzhou village study, the Shanghai ranking placed greater weight on metropolitan population structure and centre-periphery organization. Rui’s (3) comparison of urban villages and formal settlements in Shenzhen also connected settlement context with the spatial distribution of positive sentiment; the prominence of Population count and Distance to city center in Shanghai similarly points to the relevance of metropolitan structure, while the gender-specific rankings add a dimension not examined in that settlement comparison.

PDP and strict out-of-fold SVC revealed complementary forms of gender differentiation. Greenness shifted from negative to positive in both models, but the male curve rose more steeply and male-model contributions were larger in most grids. The male Openness PDP also increased, while 78.5% of grids had a larger absolute contribution in the female model. Distance to city center produced a median female-minus-male absolute-SVC difference of +1.451, with larger female-model contribution in 94.1% of grids. At the dimension level, the Distance to Transit contrast had Moran’s I = 0.653. Spatially varying and multiscale research supports these conditional relationships (45, 94, 95), while evidence on green exposure, commuting happiness, public-space use, and perceived safety provides mechanisms for the observed gender differences (82, 90–92, 98, 99). Zhang et al. (100) likewise showed that environmental associations with urban green-space sentiment changed with analytical scale; the contrast between regionally supported NDVI and street-level Greenness in Shanghai reinforces the need to interpret each predictor at its observed support.

### 4.3 Implications for healthy and equitable built-environment planning

Social-media-derived area-level happiness can support continuous spatial screening when it is paired with sample-support reporting and local verification. The 5D dimensions, socioeconomic context, prediction errors, and local SVCs identify candidate areas with persistent divergence or clustering. Field observation, resident surveys, and interviews can then determine which local conditions require action, and post-implementation monitoring can evaluate the response. Resident experience complements remote-sensing measures of urban form and network accessibility (101, 102), while extracting the reported reasons for emotions can organize public feedback (103). Minimum-user thresholds and transparent platform-label composition strengthen the basis for stratified public participation.

The 5D attribution, full-predictor PDPs, and variable-level SVCs show that the same environmental dimension can be driven by different indicators and spatial combinations. Planning should therefore focus on locally verified combinations of conditions. Design and Destination accessibility accounted for relatively large shares in the female model, whereas Design and Density were nearly equal in the male model. Field audits should accordingly examine accessibility in central and peripheral areas, first- and last-mile connections, visible greenery, enclosure, waterfront access, night-time safety, and supporting facilities. Research on green exposure emphasizes gender-specific evaluation of life satisfaction (91), and work on commuting happiness identifies differentiated relationships between the built environment and commuting burdens (92). Studies of park use and public facilities further show that opportunities for activity and amenities for staying should be designed together (98, 99). Evidence on women’s perceived safety also demonstrates that perceived spatial risk shapes entry, lingering, and avoidance (82, 90). Local participant ratings of streetscapes can complement model-based screening with on-site perceptual evidence (104). Following local verification, appropriate responses may include pocket green spaces, street-interface improvements, safer night-time routes, waterfront entrances, or resting facilities.

The same-support decline in *R*^2^, clustered residuals, and spatially varying SVCs make geographic context central to model interpretation. In central areas, audits should examine combinations of high density, central accessibility, enclosure, visible greenery, ground-floor functions, and public-space use. In peripheral areas, priority should be given to longer travel distances, bus-metro connections, everyday service access, and night-time accessibility. Built-form types can guide the selection of comparable audit areas (105). Urban big data and language models can support differentiated diagnosis (106), while spatially varying methods support location-specific assessment (45). The maps therefore direct field verification toward specific locations and environmental combinations.

### 4.4 Limitations and future research

This study has four limitations. First, platform participation, posting frequency, location choice, and binary public profile labels shape the analytical sample. The continuous score requires further cross-group measurement-invariance and construct-validity testing against direct measures of life satisfaction (92), common-infrastructure comparisons among happiness indicators (48), and multimethod affective mapping (49). Marketing language, sarcasm, and exaggeration were not classified separately and may contribute residual measurement error. Second, the six-month aggregation does not resolve diurnal, weekday-weekend, or seasonal rhythms (73), and residential, workplace, daytime, and night-time activity represent different layers of gendered spatial organization (51). Third, the 500 m grid combines streetscape, remote-sensing, accessibility, and socioeconomic measures and remains sensitive to spatial scale (46, 47); green-space attribution can also vary by type and support (100). The high-density, economically developed Shanghai setting also limits direct generalization to cities with different urban forms and platform-use patterns. Fourth, correlated predictors can share SHAP attribution, and PDP and SVC describe fitted-model contrasts. Longitudinal, quasi-experimental, and direct survey designs are needed to estimate environmental effects and strengthen population-level interpretation (45, 97).

## 5 Conclusion

This study combined geotagged UGC, 36 multiscale built-environment and contextual variables, sentiment classification, streetscape segmentation, XGBoost, same-support geographic validation, test-set SHAP, PDP, and out-of-fold SVC to examine female- and male-labelled area-level happiness in Shanghai. Three conclusions follow.

(1) Female- and male-labelled area-level happiness differed in magnitude and spatial distribution. Grid-level correspondence was modest (*r* = 0.325; *ρ* = 0.273), Moran’s I for the standardized female-minus-male contrast was 0.913, and 6.03% of grids occupied opposite extreme quartiles.
(2) Built-environment attribution differed across platform-label groups. Design ranked first in both models, Destination accessibility was more prominent in the female-labelled model, and Density approached Design in the male-labelled model. The two models shared the same dominant dimension in 43.3% of grids, while all five dimension-level differences showed significant spatial clustering. Same-support validation confirmed positive predictive performance under buffered spatial transfer, and minimum-user sensitivity remained positive through the 20-user threshold.
(3) Combining area-level happiness surfaces with local environmental contributions identifies contiguous areas where differences between the two platform-label groups are most pronounced. These areas provide priorities for resident engagement and site investigation focused on visible greenery, enclosure, waterfront access, safety, transport connections, and facility provision. The GeoXAI framework connects citywide nonlinearity with local spatial interpretation and can be recalibrated for gender-responsive planning in other data-rich cities.

## Data Availability

All de-identified grid-level data supporting the findings are available within the paper and its Supplementary information. Supplementary Data S1 contains the 500 m analytical dataset, Supplementary Data S2 contains the complete PDP and SVC result tables, and Supplementary Data S3 contains the validation, sensitivity, and out-of-fold prediction outputs. Raw Weibo post text and user-level records are not redistributed to protect user privacy and respect platform restrictions.

## Glossary

AOI: area of interest
BCR: building coverage ratio
CI: confidence interval
CNN: convolutional neural network
FAR: floor area ratio
GIS: geographic information system
HRNet: high-resolution network
LISA: local indicators of spatial association
LSTM: long short-term memory
MAE: mean absolute error
NDVI: normalized difference vegetation index
PDP: partial dependence plot
POI: point of interest
*R* ^2^: coefficient of determination
RMSE: root mean square error
RND: road network density
sDNA: spatial design network analysis
SHAP: SHapley additive exPlanations
SVC: spatially varying contribution
UGC: user-generated content
VIF: variance inflation factor
XGBoost: Extreme Gradient Boosting
