# Supplementary material for: Using GeoXAI to reveal gender differences in the spatially nonstationary influence of built environment on area-level happiness
Source: Front Public Health. 2026 Oct 14;14:1962036. doi: 10.3389/fpubh.2026.1962036 (PMC13713489; doi:10.3389/fpubh.2026.1962036)
Supplement: Supplementary file 1 [file Data_Sheet_1.PDF]

Supplementary Material

Gendered Area-Level Happiness: Using GeoXAI to Reveal Gender Differences in the Spatially Nonstationary Influence of Shanghai's Built Environment on Happiness

Cuicui Ye and Zheng Ding

These Supplementary Materials provide predictor definitions, spatial diagnostics, model-performance checks, SHAP stability analyses, spatially out-of-fold contrasts, and the complete 5D PDP-SVC panels cited in the main text.

Table S1. Predictor definitions, sources, and observed spatial support.

Predictor	Domain	Upstream source/workflow	Realised support	Definition/calculation
Visual entropy	Diversity	Baidu street-view images (March 2025); HRNet semantic segmentation	Road samples → 500 m grid	Mean image entropy; higher values indicate greater visual heterogeneity.
Color complexity	Diversity	Baidu street-view images (March 2025); HRNet semantic segmentation	Road samples → 500 m grid	Colour-distribution complexity statistic aggregated across sampled views.
Population count	Density	Socioeconomic/contextual GIS layer	17 repeated regional values	Population-count indicator aggregated at the available source geography (17 unique values in the analytical grid).
Bus density	Distance to Transit	Baidu Maps and/or OSM; GIS aggregation	500 m grid	Bus-route length divided by grid area.
Rail transit density	Distance to Transit	Baidu Maps and/or OSM; GIS aggregation	500 m grid	Rail-transit route length divided by grid area.
RND	Density	OSM road network; GIS	500 m grid	Total mapped road length divided by grid area.
FAR	Density	Baidu building AOI; GIS	500 m grid	Total gross building-floor area divided by grid area.
Gini coefficient	Socioeconomic control	Socioeconomic/contextual GIS layer	17 repeated regional values	Inequality indicator aggregated at the available regional source geography.
Housing price	Socioeconomic control	Residential-price GIS layer	500 m grid	Mean residential-market price per square metre within the grid.
Population density	Density	Population GIS layer	500 m grid	Population divided by grid area.
Residence (POIs)	Diversity	Baidu Maps POI; GIS	500 m grid	Residential POI count per square kilometre.
Commercial (POIs)	Diversity	Baidu Maps POI; GIS	500 m grid	Commercial POI count per square kilometre.
Green space (POIs)	Diversity	Baidu Maps POI; GIS	500 m grid	Green-space POI count per square kilometre.
Transportation facilities (POIs)	Diversity	Baidu Maps POI; GIS	500 m grid	Transport-facility POI count per square kilometre.
Public service (POIs)	Diversity	Baidu Maps POI; GIS	500 m grid	Public-service POI count per square kilometre.
BCR	Density	Baidu building AOI; GIS	500 m grid	Total building-footprint area divided by grid area.
Industrial (POIs)	Diversity	Baidu Maps POI; GIS	500 m grid	Industrial POI count per square kilometre.
PavingDegree	Design	Baidu street-view images (March 2025); HRNet semantic segmentation	Road samples → 500 m grid	Proportion of pixels assigned to the paving/ground-surface category.
Openness	Design	Baidu street-view images (March 2025); HRNet semantic segmentation	Road samples → 500 m grid	Proportion of sky pixels among classified image pixels.
NDVI	Design	Google Earth Engine, nominal 30 m product	17 repeated regional values in analysed table	Normalised Difference Vegetation Index. The realised 17-value support, not nominal raster resolution, governs interpretation.
Motor vehicle diversity	Diversity	Baidu street-view images (March 2025); HRNet semantic segmentation	Road samples → 500 m grid	Diversity score based on the number of detected vehicle categories.
Motor vehicle density	Density	Baidu street-view images (March 2025); HRNet semantic segmentation	Road samples → 500 m grid	Mean detected motor-vehicle count per sampled street-view image.
LUM	Diversity	Baidu Maps POI/land-use categories; GIS	500 m grid	Entropy-based land-use mix index calculated from category shares.
Greenness	Design	Baidu street-view images (March 2025); HRNet semantic segmentation	19,093 unique local values	Proportion of visible vegetation/green-ground pixels among classified pixels.
Enclosure	Design	Baidu street-view images (March 2025); HRNet semantic segmentation	Road samples → 500 m grid	Proportion assigned to the enclosure-related semantic category and aggregated across sampled views.
BtA400	Destination accessibility	OSM road network; sDNA	Street network, 400 m radius → grid	Network betweenness centrality within a 400 m radius.
BtA8000	Destination accessibility	OSM road network; sDNA	Street network, 8,000 m radius → grid	Network betweenness centrality within an 8,000 m radius.
Aquatic rate	Design	Baidu street-view images (March 2025); HRNet semantic segmentation	Road samples → 500 m grid	Proportion of water pixels among classified image pixels.

Predictor	Domain	Upstream source/workflow	Realised support	Definition/calculation
Distance to nearest bus station	Distance to Transit	Baidu Maps transit points + OSM network; GIS	500 m-grid representative location	Shortest road-network distance to the nearest bus station.
Distance to city centre	Destination accessibility	GIS	500 m-grid representative location	Road-network distance to the prespecified city-centre reference point.
Distance to nearest commerce	Destination accessibility	Baidu Maps POI + OSM network; GIS	500 m-grid representative location	Shortest road-network distance to the nearest commercial destination.
Distance to nearest green space	Destination accessibility	Baidu Maps POI + OSM network; GIS	500 m-grid representative location	Shortest road-network distance to the nearest green-space destination.
Distance to nearest transportation	Destination accessibility	Baidu Maps POI + OSM network; GIS	500 m-grid representative location	Shortest road-network distance to the nearest transport-facility destination.
Distance to nearest residence	Destination accessibility	Baidu Maps POI + OSM network; GIS	500 m-grid representative location	Shortest road-network distance to the nearest residential destination.
Distance to nearest subway	Distance to Transit	Baidu Maps transit points + OSM network; GIS	500 m-grid representative location	Shortest road-network distance to the nearest subway station.
Distance to nearest industry	Destination accessibility	Baidu Maps POI + OSM network; GIS	500 m-grid representative location	Shortest road-network distance to the nearest industrial destination.

The analysis used 33,591 grids and 36 predictors: 34 built-environment indicators were assigned to the 5D framework, while the Gini coefficient and Housing price represented socioeconomic context.

Table S2. Global Moran's I for observed outcomes and predictors.

Variable	Moran's I	Z-score	P-value
Female area-level happiness	0.909	—	0.001
Male area-level happiness	0.666	—	0.001
BtA400	0.305	78.824	<0.001
BtA8000	0.203	52.252	<0.001
LUM	0.610	155.422	<0.001
NDVI	0.678	172.563	<0.001
Housing price	0.689	175.622	<0.001
Gini coefficient	0.735	187.346	<0.001
BCR	0.751	191.277	<0.001
RND	0.684	174.165	<0.001
FAR	0.710	180.955	<0.001
Bus Density	0.607	154.719	<0.001
Rail transit density	0.488	124.384	<0.001
Population density	0.349	89.998	<0.001
Motor vehicle diversity	0.712	181.454	<0.001
Motor vehicle density	0.696	177.404	<0.001
Commerce (POIs)	0.556	141.849	<0.001
Industry (POIs)	0.560	142.730	<0.001
Green space (POIs)	0.308	79.177	<0.001
Public service (POIs)	0.715	182.454	<0.001
Residence (POIs)	0.514	131.511	<0.001
Transportation service (POIs)	0.666	169.717	<0.001
Distance to the nearest subway station	1.002	255.321	<0.001
Distance to the nearest industry	1.000	254.876	<0.001
Distance to the nearest transportation	1.000	254.761	<0.001
Distance to the nearest residence	0.999	254.495	<0.001
Distance to the nearest green space	0.988	251.696	<0.001
Distance to the nearest commerce	0.999	254.626	<0.001
Distance to the nearest bus station	1.000	254.862	<0.001
Distance to city center	1.000	254.675	<0.001
Openness	0.634	161.399	<0.001
Greenness	0.574	146.371	<0.001
Paving degree	0.687	174.974	<0.001
Population count	0.697	177.550	<0.001
Aquatic rate	0.412	104.944	<0.001
Enclosure	0.669	170.508	<0.001
Color complexity	0.697	177.550	<0.001

Variable	Moran’s I	Z-score	P-value
Visual entropy	0.706	179.965	<0.001

The two happiness surfaces were evaluated on the 500 m grid using binary Rook contiguity and 999 permutations. The remaining rows report the same spatial-autocorrelation diagnostics for the predictors.

Table S3. Out-of-fold performance, fold variability, and calibration.

Outcome	Validation	n	Pooled OOF R^2	Fold R^2 range	RMSE	MAE	Calibration intercept	Calibration slope	Mean bias
Male happiness	Random five-fold	33,591	0.930	0.880–0.948	0.451	0.214	-0.012	1.004	-0.001
Male happiness	Coordinate-group five-fold	33,591	0.882	0.816–0.913	0.586	0.320	-0.043	1.006	0.025
Female happiness	Random five-fold	33,591	0.898	0.896–0.900	0.681	0.306	-0.047	1.012	-0.001
Female happiness	Coordinate-group five-fold	33,591	0.759	0.343–0.873	1.046	0.511	0.233	0.952	-0.038

Table S4. Stability of leading random-fold out-of-fold SHAP rankings.

Outcome	Feature	Aggregate rank	Mean |SHAP|	Share of total	Role in analysis	Predictor set	Interpretation
Female happiness	NDVI	1	0.673	21.2%	Design	36 original predictors	Random-fold OOF model attribution
Female happiness	Gini coefficient	2	0.564	17.8%	Contextual control	36 original predictors	Random-fold OOF model attribution
Female happiness	Population count	3	0.411	13.0%	Density	36 original predictors	Random-fold OOF model attribution
Female happiness	Greenness	4	0.318	10.0%	Design	36 original predictors	Random-fold OOF model attribution
Female happiness	Distance to city center	5	0.307	9.7%	Distance to transit/destinations	36 original predictors	Random-fold OOF model attribution
Male happiness	Population count	1	0.647	21.4%	Density	36 original predictors	Random-fold OOF model attribution
Male happiness	Greenness	2	0.621	20.5%	Design	36 original predictors	Random-fold OOF model attribution
Male happiness	Gini coefficient	3	0.318	10.5%	Contextual control	36 original predictors	Random-fold OOF model attribution
Male happiness	Distance to city center	4	0.244	8.1%	Distance to transit/destinations	36 original predictors	Random-fold OOF model attribution
Male happiness	NDVI	5	0.171	5.6%	Design	36 original predictors	Random-fold OOF model attribution

Table S5. Strict spatial out-of-fold SVC summary.

Outcome	Rank	Feature	P95 |raw SVC|	Mean |raw SVC|	Median raw SVC	Positive	Negative	Normalised spatial SD
Female happiness	1	Distance to city center	3.359	1.932	1.972	97.82%	2.18%	0.273
Female happiness	2	RND	1.868	0.657	-0.448	5.22%	94.78%	0.293
Female happiness	3	Motor vehicle density	0.796	0.288	-0.222	6.86%	93.14%	0.282
Female happiness	4	Greenness	0.784	0.297	0.213	85.68%	14.32%	0.336
Female happiness	5	PavingDegree	0.388	0.129	-0.018	44.72%	55.28%	0.398
Female happiness	6	Aquatic rate	0.535	0.229	-0.205	8.86%	91.14%	0.304
Female happiness	7	Visual entropy	0.561	0.190	0.114	77.20%	22.80%	0.344
Female happiness	8	FAR	0.668	0.268	-0.195	21.18%	78.82%	0.372
Female happiness	9	Enclosure	0.435	0.153	-0.113	10.77%	89.23%	0.299
Female happiness	10	Openness	0.485	0.227	-0.203	11.20%	88.80%	0.341
Male happiness	1	Distance to city center	1.256	0.493	0.097	55.20%	44.80%	0.454
Male happiness	2	Greenness	0.754	0.422	0.409	99.43%	0.57%	0.231
Male happiness	3	Population density	0.587	0.369	-0.366	0.17%	99.83%	0.207

Outcome	Rank	Feature	P95 |raw SVC|	Mean |raw SVC|	Median raw SVC	Positive	Negative	Normalised spatial SD
Male happiness	4	Distance to the nearest residence	0.878	0.296	-0.202	16.02%	83.98%	0.316
Male happiness	5	Industrial (POIs)	0.602	0.243	-0.203	2.97%	97.03%	0.273
Male happiness	6	Enclosure	0.513	0.254	0.243	97.33%	2.67%	0.275
Male happiness	7	Aquatic rate	0.352	0.160	0.144	95.31%	4.69%	0.284
Male happiness	8	PavingDegree	0.635	0.267	0.206	88.87%	11.13%	0.353
Male happiness	9	Visual entropy	0.389	0.124	0.075	79.54%	20.46%	0.325
Male happiness	10	FAR	0.402	0.163	-0.136	12.03%	87.97%	0.320

Table S6. Spatial concordance and differentiation between female and male area-level happiness.

Layer	Metric	Value	Support/interpretation
Support	Analytical grids	33,591	Common female- and male-labelled outcome support
Support	Binary Rook neighbours	66,378	Undirected pairs; 132,756 directed entries; no islands
Observed surface	Female happiness Moran's I	0.909 (p = 0.001)	999 two-sided permutations
Observed surface	Male happiness Moran's I	0.666 (p = 0.001)	999 two-sided permutations
OOF residual	Female happiness Moran's I	0.699 (p = 0.001)	Coordinate-group OOF residual
OOF residual	Male happiness Moran's I	0.502 (p = 0.001)	Coordinate-group OOF residual
Same-grid concordance	Pearson r	0.325	Descriptive continuous-score concordance
Same-grid concordance	Spearman rho	0.273	Descriptive rank concordance
Raw score contrast	Mean female minus male	+1.115	Difference across matched analytical grids
Raw score contrast	Median female minus male	+1.060	Difference across matched analytical grids
Spatial contrast	Raw-gap Moran's I	0.948 (p = 0.001)	999 two-sided permutations
Spatial contrast	Standardised-gap Moran's I	0.913 (p = 0.001)	Primary relative-position contrast z(F)-z(M)
Cross-surface	Symmetric cross-surface Moran statistic	0.171 (pseudo-p = 0.001)	Defined in Methods; neighbouring labelled surfaces
Extreme overlap	High-quartile Jaccard	0.279	Within-outcome 75th-percentile sets
Extreme overlap	Low-quartile Jaccard	0.208	Within-outcome 25th-percentile sets
Extreme co-location	Both high	10.90%	3,663 grids
Extreme co-location	Both low	8.62%	2,897 grids
Extreme co-location	Female high / Male low	3.67%	1,234 grids
Extreme co-location	Male high / Female low	2.36%	793 grids
Local standardised gap	BH-adjusted High-High	4,137	Conditional-randomisation Local Moran q < 0.05
Local standardised gap	BH-adjusted Low-Low	3,636	Conditional-randomisation Local Moran q < 0.05
Local standardised gap	BH-adjusted local outliers	2	High-Low = 1; Low-High = 1

Table S7. 5D predictors, SHAP ranks, and spatially out-of-fold SVC summaries.

5D dimension	Predictor	Main-model SHAP rank	Spatial-contrast rule	Within-dimension SVC rank	Female mean |SVC|	Male mean |SVC|
Density	RND	2	Interquartile	1	0.6504	0.1658
Density	Population density	3	Interquartile	2	0.1255	0.3647
Density	FAR	5	Interquartile	3	0.2734	0.1662
Density	Motor vehicle density	6	Interquartile	4	0.2913	0.0999
Diversity	Industrial (POIs)	4	Interquartile	1	0.1902	0.2411
Diversity	Visual entropy	3	Interquartile	2	0.1951	0.1223
Diversity	Public service (POIs)	7	Interquartile	3	0.0530	0.0314
Diversity	Transportation facilities (POIs)	6	Interquartile	4	0.0223	0.0616
Diversity	LUM	5	Interquartile	5	0.0489	0.0325
Diversity	Motor vehicle diversity	1	Interquartile	6	0.0434	0.0218
Diversity	Residence (POIs)	9	Interquartile	7	0.0167	0.0180
Diversity	Green space (POIs)	10	Adaptive empirical quantiles	8	0.0037	0.0020
Design	Greenness	1	Interquartile	1	0.2944	0.4194
Design	Enclosure	4	Interquartile	2	0.1430	0.2583
Design	Aquatic rate	6	Interquartile	3	0.2382	0.1584
Design	PavingDegree	5	Interquartile	4	0.1204	0.2657
Design	Openness	3	Interquartile	5	0.2119	0.0944

5D dimension	Predictor	Main-model SHAP rank	Spatial-contrast rule	Within-dimension SVC rank	Female mean |SVC|	Male mean |SVC|
Destination accessibility	Distance to city center	1	Interquartile	1	1.9273	0.4897
Destination accessibility	Distance to the nearest residence	2	Interquartile	2	0.2058	0.3023
Destination accessibility	Distance to the nearest green space	5	Interquartile	3	0.1806	0.0484
Destination accessibility	BtA400	8	Interquartile	4	0.1289	0.0780
Destination accessibility	BtA8000	6	Interquartile	5	0.1330	0.0648
Destination accessibility	Distance to the nearest commerce	3	Interquartile	6	0.0400	0.0260
Destination accessibility	Distance to the nearest transportation	4	Interquartile	7	0.0231	0.0344
Destination accessibility	Distance to the nearest industry	7	Interquartile	8	0.0302	0.0184
Distance to Transit	Bus density	4	Interquartile	1	0.1311	0.0647
Distance to Transit	Rail transit density	3	Adaptive empirical quantiles	2	0.1450	0.0418
Distance to Transit	Distance to the nearest subway	2	Interquartile	3	0.0597	0.0420
Distance to Transit	Distance to the nearest bus station	1	Interquartile	4	0.0663	0.0310

Rail transit density and Green space (POIs) use adaptive empirical-quantile contrasts because their interquartile contrasts collapse locally; all other predictors use the outer-training interquartile contrast.

Table S8. Fold-to-fold uncertainty of the PDP curves for the 31 indicators in the paired PDP-SVC comparison.

5D dimension	Rank	Predictor	Model	Median interval width	Maximum interval width	Maximum |full PDP|	Fold models
Density	1	Population count	Female happiness	0.0306	0.1329	0.9822	5
Density	1	Population count	Male happiness	0.0716	0.1141	1.0259	5
Density	2	RND	Female happiness	0.0467	0.1180	0.3696	5
Density	2	RND	Male happiness	0.0323	0.0721	0.3015	5
Density	3	Population density	Female happiness	0.0084	0.1495	0.2523	5
Density	3	Population density	Male happiness	0.0121	0.0792	0.4280	5
Density	4	FAR	Female happiness	0.0564	0.1004	0.0482	5
Density	4	FAR	Male happiness	0.0393	0.1929	0.2325	5
Density	5	Motor vehicle density	Female happiness	0.0081	0.0958	0.0436	5
Density	5	Motor vehicle density	Male happiness	0.0114	0.0555	0.0501	5
Diversity	1	Motor vehicle diversity	Female happiness	0.0137	0.1670	0.1495	5
Diversity	1	Motor vehicle diversity	Male happiness	0.0888	0.0901	0.2427	5
Diversity	2	Visual entropy	Female happiness	0.0177	0.0797	0.0379	5
Diversity	2	Visual entropy	Male happiness	0.0095	0.0191	0.0224	5
Diversity	3	Industrial (POIs)	Female happiness	0.0463	0.0543	0.0396	5
Diversity	3	Industrial (POIs)	Male happiness	0.0359	0.0420	0.1853	5
Diversity	4	LUM	Female happiness	0.0138	0.0299	0.0533	5
Diversity	4	LUM	Male happiness	0.0052	0.0338	0.0132	5
Diversity	5	Transportation facilities (POIs)	Female happiness	0.0243	0.0747	0.0563	5
Diversity	5	Transportation facilities (POIs)	Male happiness	0.0069	0.0924	0.0303	5
Diversity	6	Public service (POIs)	Female happiness	0.0451	0.0470	0.0268	5
Diversity	6	Public service (POIs)	Male happiness	0.0234	0.0769	0.0337	5
Diversity	7	Residence (POIs)	Female happiness	0.0042	0.0079	0.0037	5
Diversity	7	Residence (POIs)	Male happiness	0.0321	0.1067	0.1044	5

5D dimension	Rank	Predictor	Model	Median interval width	Maximum interval width	Maximum |full PDP|	Fold models
Diversity	8	Green space (POIs)	Female happiness	0.0054	0.0070	0.0046	5
Diversity	8	Green space (POIs)	Male happiness	0.0089	0.0300	0.0123	5
Design	1	Greenness	Female happiness	0.0263	0.1239	0.4126	5
Design	1	Greenness	Male happiness	0.0840	0.1331	1.1216	5
Design	2	NDVI	Female happiness	0.0455	0.0813	0.7955	5
Design	2	NDVI	Male happiness	0.0262	0.0456	0.3356	5
Design	3	Openness	Female happiness	0.0994	0.1425	0.0893	5
Design	3	Openness	Male happiness	0.0589	0.0816	0.3234	5
Design	4	Enclosure	Female happiness	0.0292	0.1106	0.0420	5
Design	4	Enclosure	Male happiness	0.0270	0.0532	0.0353	5
Design	5	PavingDegree	Female happiness	0.0227	0.0573	0.0212	5
Design	5	PavingDegree	Male happiness	0.0145	0.0461	0.0577	5
Design	6	Aquatic rate	Female happiness	0.0076	0.0260	0.0083	5
Design	6	Aquatic rate	Male happiness	0.0076	0.0676	0.0245	5
Destination accessibility	1	Distance to city center	Female happiness	0.0467	0.2213	1.0038	5
Destination accessibility	1	Distance to city center	Male happiness	0.0184	0.2351	0.5594	5
Destination accessibility	2	Distance to the nearest residence	Female happiness	0.0202	0.1996	0.4982	5
Destination accessibility	2	Distance to the nearest residence	Male happiness	0.0330	0.0969	0.1580	5
Destination accessibility	3	Distance to the nearest commerce	Female happiness	0.0458	0.5517	0.7483	5
Destination accessibility	3	Distance to the nearest commerce	Male happiness	0.0418	0.0699	0.1583	5
Destination accessibility	4	Distance to the nearest transportation	Female happiness	0.0596	0.3111	0.1968	5
Destination accessibility	4	Distance to the nearest transportation	Male happiness	0.0312	0.2005	0.1066	5
Destination accessibility	5	Distance to the nearest green space	Female happiness	0.0190	0.0730	0.2858	5
Destination accessibility	5	Distance to the nearest green space	Male happiness	0.0109	0.1171	0.1623	5
Destination accessibility	6	BtA8000	Female happiness	0.0289	0.0658	0.0866	5
Destination accessibility	6	BtA8000	Male happiness	0.0314	0.0620	0.0225	5
Destination accessibility	7	Distance to the nearest industry	Female happiness	0.0157	0.0645	0.1166	5
Destination accessibility	7	Distance to the nearest industry	Male happiness	0.0061	0.0658	0.0452	5
Destination accessibility	8	BtA400	Female happiness	0.0079	0.0122	0.0044	5
Destination accessibility	8	BtA400	Male happiness	0.0115	0.0641	0.0177	5
Distance to Transit	1	Distance to the nearest bus station	Female happiness	0.0480	0.3427	0.0612	5
Distance to Transit	1	Distance to the nearest bus station	Male happiness	0.0258	0.1666	0.0792	5
Distance to Transit	2	Distance to the nearest subway	Female happiness	0.0139	0.0634	0.0660	5
Distance to Transit	2	Distance to the nearest subway	Male happiness	0.0079	0.0259	0.0234	5
Distance to Transit	3	Rail transit density	Female happiness	0.1580	0.2557	0.0423	5
Distance to Transit	3	Rail transit density	Male happiness	0.0076	0.1326	0.1729	5
Distance to Transit	4	Bus density	Female happiness	0.0049	0.0284	0.0056	5
Distance to Transit	4	Bus density	Male happiness	0.0081	0.0208	0.0122	5

Intervals are the 2.5th-97.5th percentile range across five random-fold training models evaluated on a common predictor grid.

Figure S1. Random-fold and coordinate-group performance, calibration, fold-level R^2 variability, and random-fold out-of-fold SHAP rank stability.

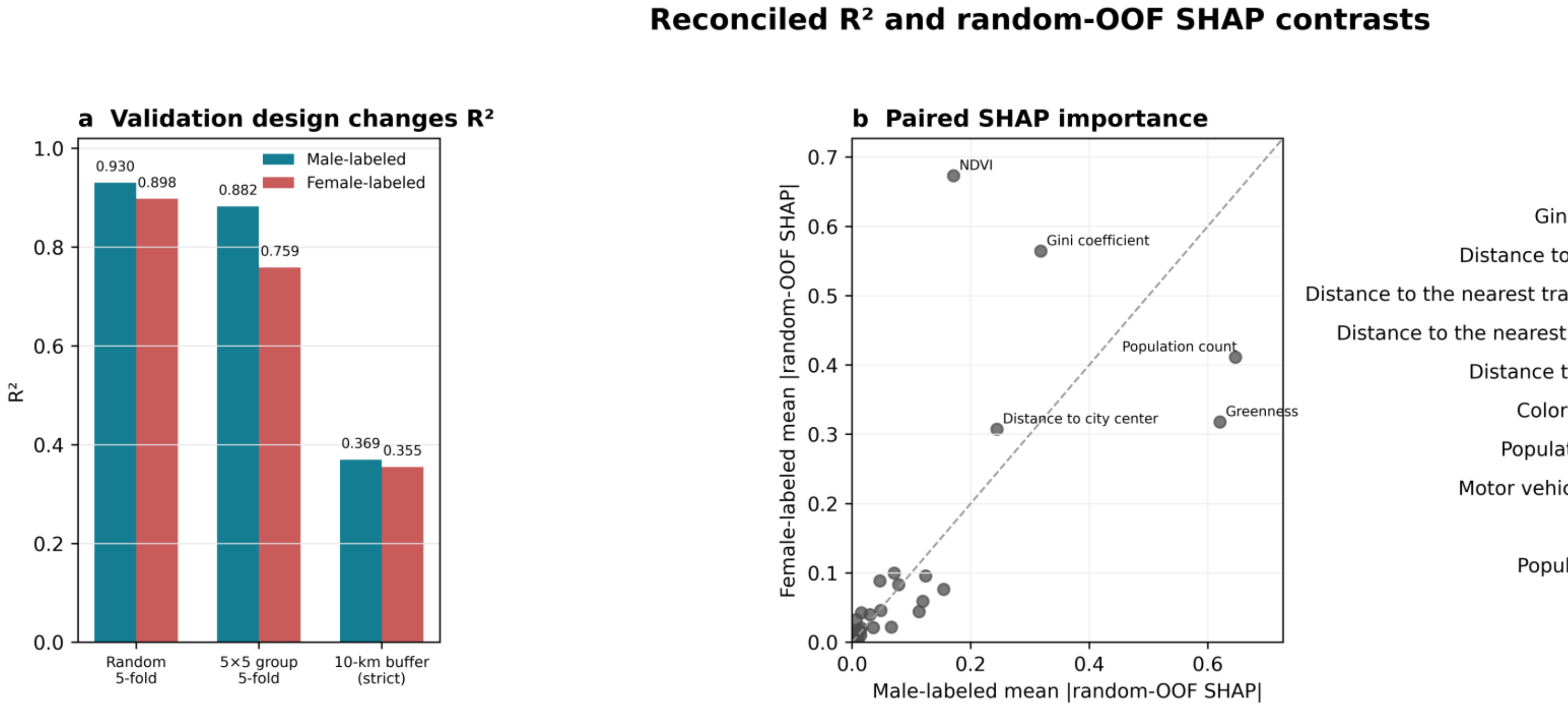

Figure S2. Density indicators and socioeconomic contextual variables: paired global PDP and local female-minus-male differences in absolute SVC.

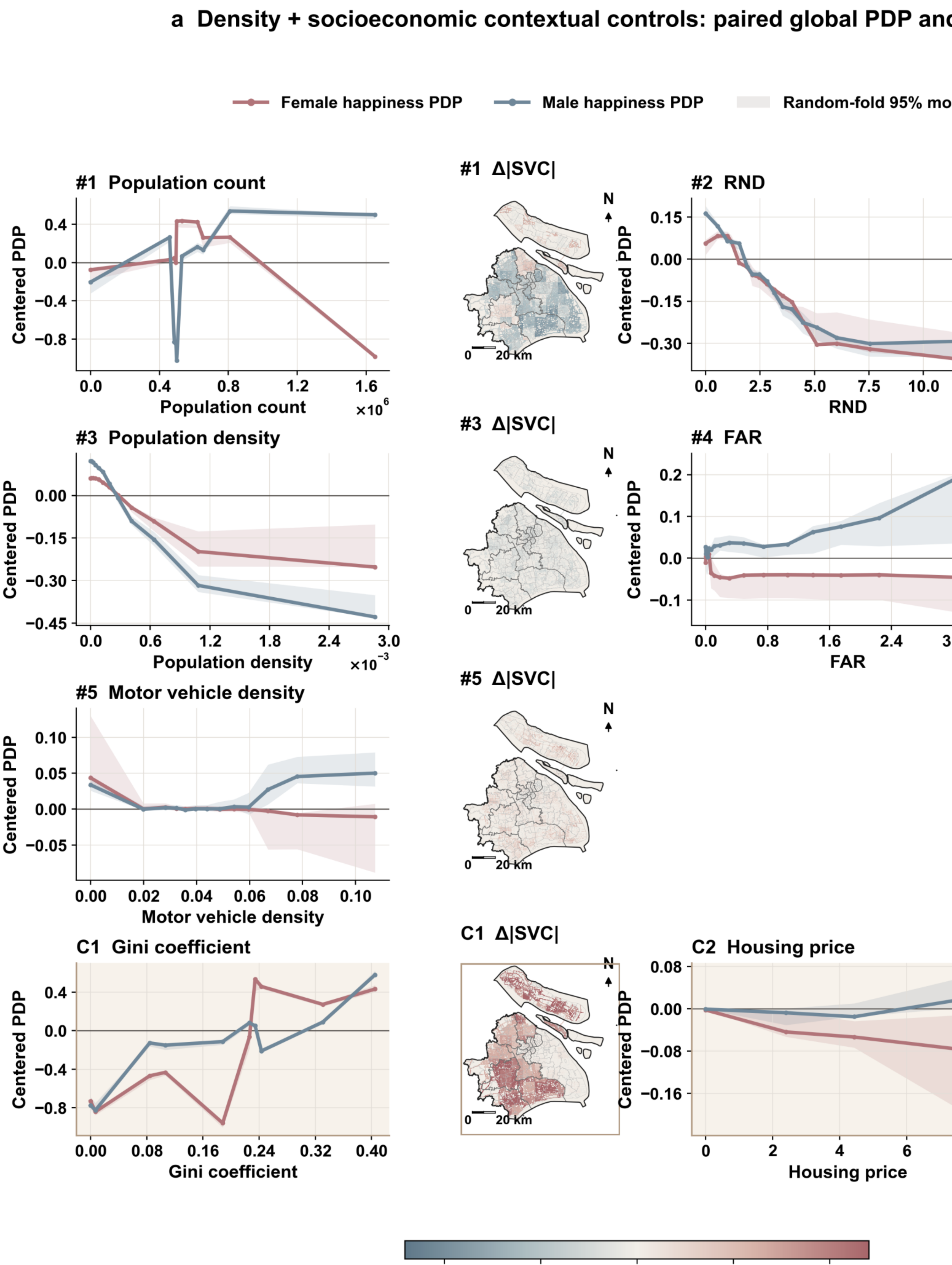
a Density + socioeconomic contextual controls: paired global PDP anc
Female happiness PDP
Male happiness PDP
Random-fold 95% mo
#1 Population count
Centered PDP
Population count
×10^6
#1 Δ|SVC|
N
0
20 km
#2 RND
RND
#3 Population density
Population density
×10^-3
#3 Δ|SVC|
#4 FAR
FAR
#5 Motor vehicle density
Motor vehicle density
#5 Δ|SVC|
C1 Gini coefficient
Gini coefficient
C1 Δ|SVC|
C2 Housing price
Housing price

Figure S3. Diversity indicators: paired global PDP and local female-minus-male differences in absolute SVC.

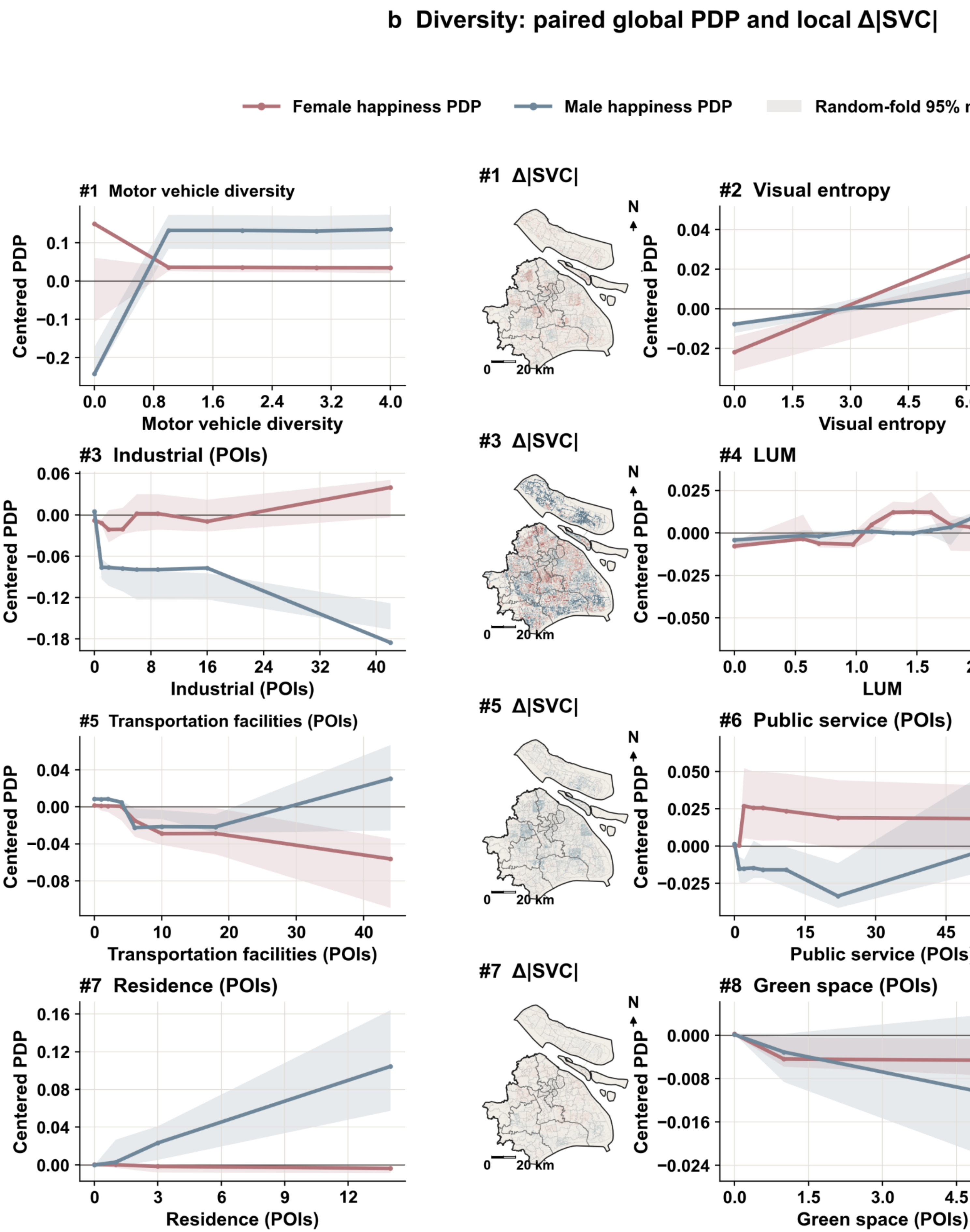
b Diversity: paired global PDP and local Δ|SVC|
Female happiness PDP
Male happiness PDP
#1 Motor vehicle diversity
Centered PDP
Motor vehicle diversity
#1 Δ|SVC|
N
0
20 km
#2 Visual entropy
Visual entropy
#3 Industrial (POIs)
Industrial (POIs)
#3 Δ|SVC|
#4 LUM
LUM
#5 Transportation facilities (POIs)
Transportation facilities (POIs)
#5 Δ|SVC|
#6 Public service (POIs)
#7 Residence (POIs)
Residence (POIs)
#7 Δ|SVC|
#8 Green space (POIs)
Green space (POIs)

Figure S4. Design indicators: paired global PDP and local female-minus-male differences in absolute SVC.

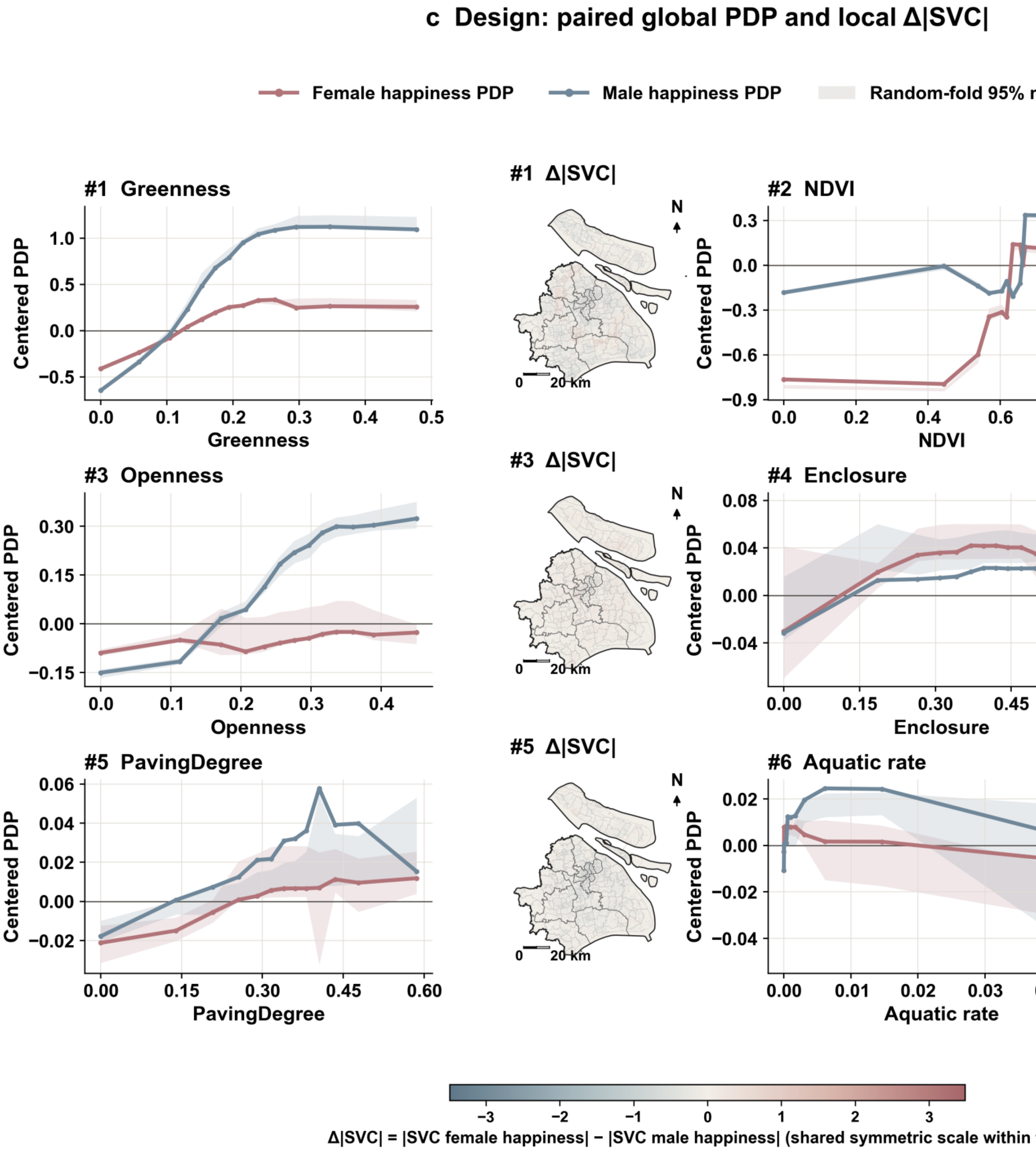

Figure S5. Destination-accessibility indicators: paired global PDP and local female-minus-male differences in absolute SVC.

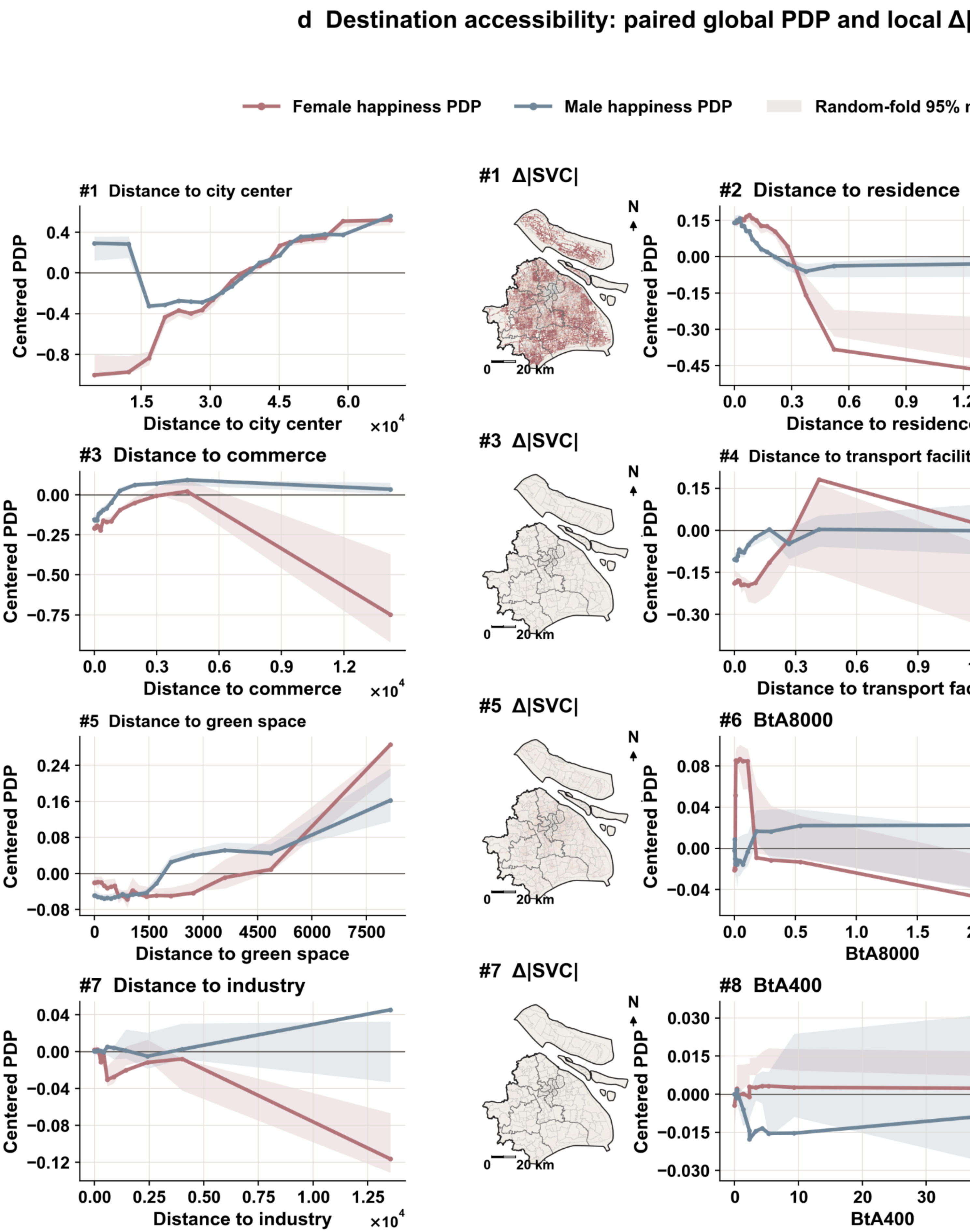
d Destination accessibility: paired global PDP and local Δ|
Female happiness PDP
Male happiness PDP
Random-fold 95% r
#1 Distance to city center
Centered PDP
Distance to city center
×10^4
#1 Δ|SVC|
N
0
20 km
#2 Distance to residence
Distance to residence
#3 Distance to commerce
Distance to commerce
#3 Δ|SVC|
#4 Distance to transport facilit
Distance to transport fa
#5 Distance to green space
Distance to green space
#5 Δ|SVC|
#6 BtA8000
BtA8000
#7 Distance to industry
Distance to industry
#7 Δ|SVC|
#8 BtA400
BtA400

Figure S6. Distance-to-Transit indicators: paired global PDP and local female-minus-male differences in absolute SVC.

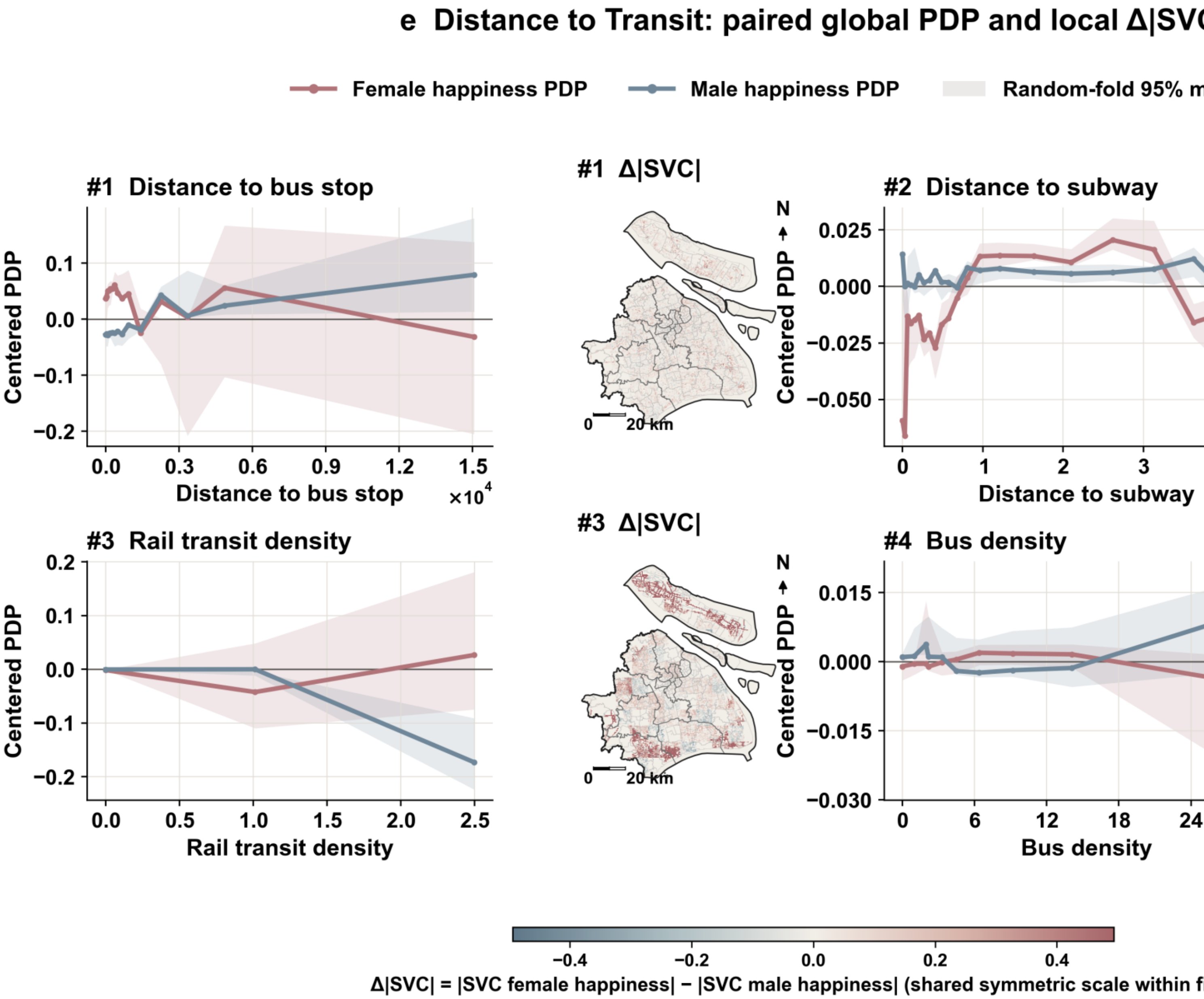

Figure S7. Female-minus-male differences in strict spatial out-of-fold SVC intensity across the five 5D dimensions.

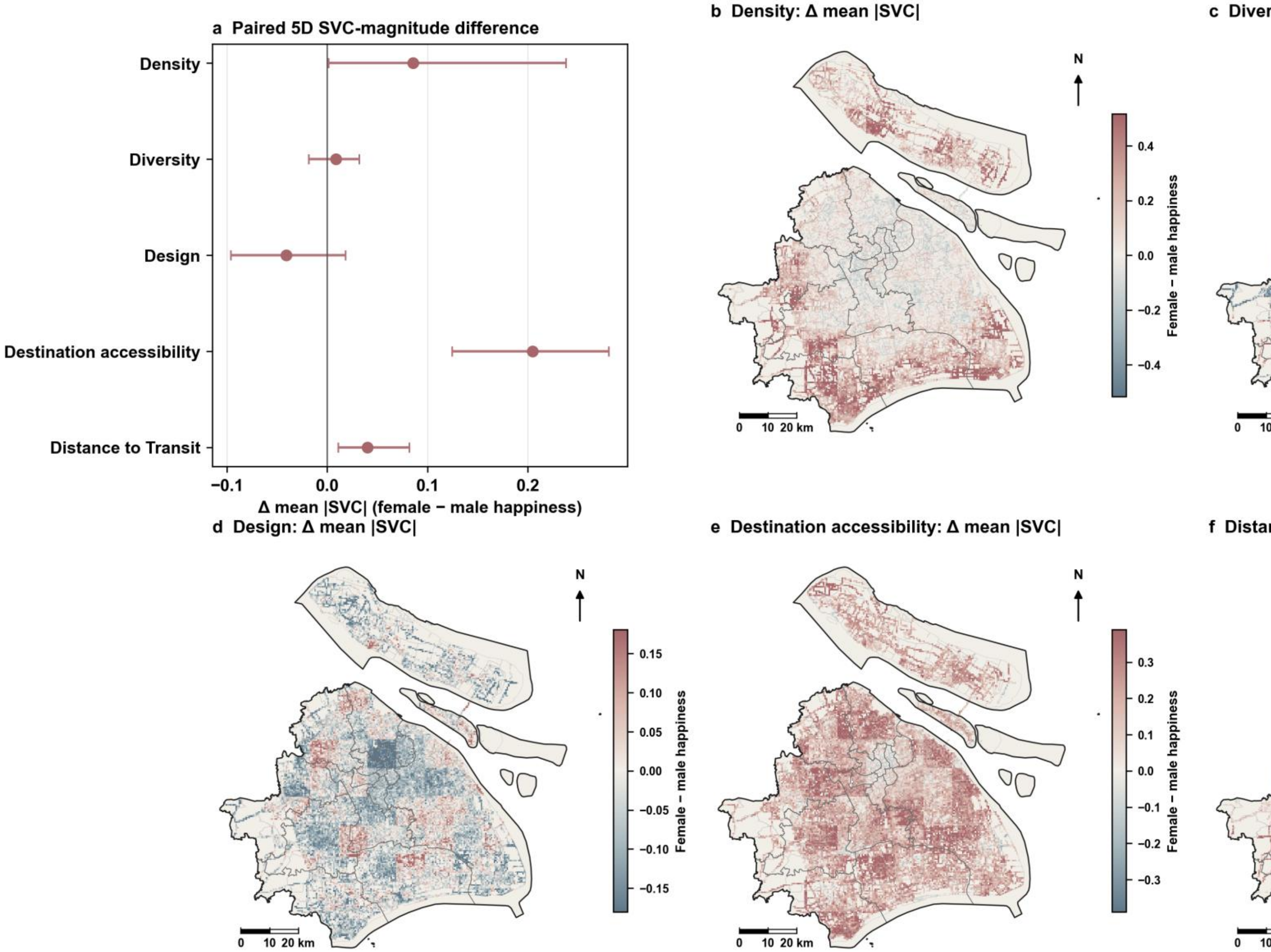

Additional data files

Additional file 2 provides the de-identified 500 m-grid analytical dataset. Additional file 3 provides the PDP and SVC summary tables used in the main and supplementary figures.
