# Supplementary material for: Using GeoXAI to reveal gender differences in the spatially nonstationary influence of built environment on area-level happiness
Source: Front Public Health. 2026 Oct 14;14:1962036. doi: 10.3389/fpubh.2026.1962036 (PMC13713489; doi:10.3389/fpubh.2026.1962036)
Supplement: Supplementary file 3 [file Data_Sheet_5.PDF]

Response to Reviewers

Manuscript ID: 1962036

Revised title: Using GeoXAI to Reveal Gender Differences in the Spatially Nonstationary Influence of Built Environment on Area-Level Happiness

We thank the editor and both reviewers for their careful reading. We revised the manuscript point by point and provide the responses below. The revised submission includes a marked manuscript, a clean manuscript, Supplementary Material, and three supporting datasets. Supplementary Data S1 contains the de-identified 500 m grid dataset, Supplementary Data S2 contains the PDP and SVC result tables, and Supplementary Data S3 contains the sensitivity and model-comparison tables, out-of-fold predictions, figure-source data, and reproducibility manifest. All manuscript changes are shown in red in the marked version. The reviewer comments below are reproduced verbatim.

Response to Reviewer 1

Reviewer 1 | 30 Aug 2026 | 02:35 | Comment #1

> ***Reviewer comment:*** *The manuscript addresses an important topic and presents a strong methodological framework integrating social-media data, street-view imagery, XGBoost, SHAP, and spatial validation. However, several issues require major revision. First, the sentiment-derived score should be described more cautiously as expressed or social-media-derived happiness, since construct validity and cross-gender measurement equivalence remain uncertain. Second, the authors should report the number of posts and unique users per 500 m grid and perform sensitivity analyses using minimum sample thresholds. Third, differences in spatial support among predictors and the large number of grids with zero streetscape indicators may influence model performance and should be further examined. In addition, the decline in R^2 under strict spatial validation should be evaluated using the same sample and predictor set to isolate the effect of geographic separation. Finally, causal terms such as "influence" and strong interpretations of "gendered spatial segregation" should be moderated. Overall, the study is promising but requires substantial methodological clarification and robustness analyses before publication.*

Response: We thank Reviewer 1 for the careful assessment and for identifying five issues that required clarification. We addressed each point in the revised manuscript.

(1) We retained the term area-level happiness but now define it explicitly as the mean sentiment-derived happiness score expressed in publicly visible, geotagged posts within each 500 m grid. The manuscript distinguishes this ecological, social-media-derived construct from individual survey measures and identifies cross-group construct validation as a future requirement (Lines 59–73, 181–207, and 665–680).

(2) We now report post and unique-user support by grid and evaluate thresholds of at least 1, 3, 5, 10, and 20 users in both platform-label groups. The Methods describe the 507,550 matched posts across 7,252 grids, the 4,832 grids containing both groups, and the median post and user counts. The corresponding sensitivity results are provided in the Results and Supporting Information (Lines 198–207, 237–244, and 404–412; Tables S9–S10; Figure S8).

(3) We report the realized spatial support of all predictors and examine the 14,478 grids with zero values across the eight streetscape indicators. A matched analysis compares the 30-predictor model with a 23-predictor non-streetscape model on the same 19,113 grids, and also reports the 23-predictor result for all 33,591 grids (Lines 147–179, 208–244, and 408–412; Tables S1 and S13; Figure S9).

(4) The decline under geographic separation is now reported on identical support. Ridge, PLS regression, KNN, Linear SVR, and XGBoost use the same 19,113 grids, 30 predictors, and random and buffered folds. For XGBoost, R^2 changed from 0.587 to 0.353 for the female-labelled outcome and from 0.568 to 0.363 for the male-labelled outcome (Lines 285–305 and 386–396; Tables S12–S14; Figure S9).

(5) Empirical findings are now described as associations, model attributions, contributions, and fitted contrasts. The Results use the term gendered spatial differentiation in area-level happiness, while the Discussion treats

segregation as a theoretical context for future mobility, residential, and population-level research rather than as a directly measured outcome (Lines 84–100, 285–337, 522–547, and 665–680).

Response to Reviewer 2

Reviewer 2 | 24 Aug 2026 | 16:53 | Comment #1

Comment 1

Reviewer comment: *1. The title needs to be more concise.*

Response: We agree and shortened the title by removing the city name and the repetitive opening phrase. The revised title is "Using GeoXAI to Reveal Gender Differences in the Spatially Nonstationary Influence of Built Environment on Area-Level Happiness" (Title page).

Comment 2(a): Abstract

Reviewer comment: *2. Abstract: "Moran's I = 0.909 and 0.666" refers to Moran's I values of 0.909 for females and 0.666 for males, respectively? Do the indicators used by the authors represent the "Built Environment"? Based on the abstract, I feel that the study places greater emphasis on regional differences rather than the effects of the Built Environment. If this is the case, both the title and the overall focus of the manuscript should be revised accordingly.*

Response: We clarified the order of the spatial statistics: Moran's I is 0.909 for the female-labelled surface and 0.666 for the male-labelled surface. The Abstract also identifies the 5D built-environment analysis, the leading predictors, and the local contribution differences, so the regional patterns are presented as part of the built-environment findings rather than as a separate focus (Lines 7–26).

Comment 2(b): Introduction

Reviewer comment: *2. Introduction: "Built Environment" needs to be clearly defined. What indicators have previous studies typically used to quantify the built environment? In addition, more discussion is needed on the effects of the Built Environment on Happiness in the existing literature. What is the main innovation of this study? Is it the use of a different research method, such as GeoXAI? Moreover, Gender Differences is one of the key research components of this study, but it is only briefly discussed in the Introduction. I therefore believe that the Introduction needs to be substantially revised, with greater emphasis on the effects of the Built Environment on Happiness and Gender Differences, as these should be the primary research objectives of the study.*

Response: The Introduction now defines the built environment through buildings, streets, transport networks, land uses, public spaces, and landscape elements, and identifies density, functional mix, accessibility, street design, and transit connections as common measurement domains. It also expands the literature on happiness and gendered mobility, care responsibilities, safety, and resource access. The final paragraph states the GeoXAI contribution through common-support validation, nonlinear interpretation, local contribution mapping, and paired gender-specific spatial diagnostics (Lines 30–100).

Comment 3

Reviewer comment: *3. Why was Shanghai selected as the study area? Please provide a clear justification for choosing Shanghai.*

Response: We expanded the rationale for Shanghai. Its high density, extensive public transport, strong centre–periphery contrasts, and mixture of historic, renewed, and rapidly urbanizing districts produce substantial variation in accessibility, streetscape quality, and socioeconomic conditions, making it an appropriate setting for testing spatially varying built-environment associations (Lines 74–83 and 102–110).

Comment 4

Reviewer comment: *4. Materials and methods: The manuscript relies on geotagged Weibo posts. While these are public, PLOS requires explicit ethics statements. Please clarify: (1) Compliance with Weibo's terms of*

service. (2) Whether data were anonymized beyond public visibility. (3) Institutional ethics review or waiver, even for public online data.

Response: We clarified that the study used publicly accessible mobile-location pages and did not bypass access controls. Direct identifiers were removed before analysis, results were aggregated to 500 m grids, and no raw post text or user-level record is reproduced or redistributed. The manuscript states that this was secondary analysis of public, de-identified material without recruitment or interaction. The available institutional record contains no ethics approval or waiver identifier, so no committee name or reference number has been introduced (Lines 181–207, 341–347, and 720–725).

Comment 5

Reviewer comment: *Materials and methods:*
5. Expand the discussion on sampling bias: The study uses data only from Shanghai. Is this sample representative? I believe the findings may only be applicable to economically developed regions, and the limited geographic scope may constrain the generalizability of the conclusions.

Response: We expanded the limitations to address platform participation, posting frequency, location choice, binary public profile labels, the six-month observation period, spatial scale, and the need for cross-group construct validation. The revised text also states that Shanghai's high-density and economically developed setting limits direct generalization to cities with different urban forms and platform-use patterns; application elsewhere requires local recalibration (Lines 665–680 and 695–700).

Comment 6

Reviewer comment: *6. Data collection: The data collection and preprocessing procedures are described too briefly. The authors only state that 678,120 geotagged data points were obtained, but do not provide sufficient details on how the data were cleaned. Specifically, how were advertisements and marketing-related posts removed? How were sarcastic or exaggerated expressions identified and excluded? How were missing values handled? Since Weibo contains a large number of reposted or duplicated posts, how did the authors identify and remove duplicate or repeated information? For transparency and reproducibility, the authors should report the number of observations removed at each data-cleaning step and the final sample size after preprocessing.*

Response: We expanded the processing description and report every count supported by the retained records: 678,120 acquired posts, 659,067 initially retained records, 659,064 normalized records, 590,513 exact cleaned-text deduplicated records, and 507,707 polarity-scored records. The raw file contained 18,948 records without a public platform-gender label, 152 records without text, and no missing coordinates; these conditions could overlap. URLs, hashtags, emojis, and non-text symbols were processed as described, exact normalized-text duplicates were removed, and texts shorter than 5 or longer than 150 characters or dominated by uninterpretable emojis or special characters were excluded before scoring. The count file linked 507,550 scored posts to 500 m grids; the remaining 157 fell just outside Shanghai's municipal boundary after coordinate alignment. Marketing language, sarcasm, and exaggeration were not classified as separate cleaning categories and are stated as a source of residual measurement error (Lines 181–207 and 665–680; Table S9).

Comment 7

Reviewer comment: *7. Multi-source urban data: The multi-source urban data should be provided in the Supporting Information (SI), together with the data source websites and detailed information on how the data were obtained. In addition, the authors should clearly specify the sources of the 36 built-environment predictors and explain how these variables were collected, processed, and quantified.*

Response: Supplementary Table S1 now lists all 36 predictors with their domain, source website where available, acquisition or derivation procedure, definition, missing-value treatment, and realized spatial support. The Methods summarize the source families, acquisition timing, projection, and aggregation to the common 500 m grid, and Supplementary Data S1 provides the de-identified grid-level analytical dataset (Lines 143–179 and 208–235; Table S1).

Comment 8

__Reviewer comment:__ 8. Sentiment analysis: Why did the authors choose the LSTM-CNN model for sentiment analysis? Is its accuracy sufficiently reliable, and was the model externally validated using an independently annotated dataset? In addition, did the authors compare LSTM-CNN with other commonly used sentiment analysis methods, such as BERT, BiLSTM, CNN, or lexicon-based approaches? A systematic comparison would help demonstrate the robustness and reliability of the selected method. Recent literature indicates that different sentiment analysis methods can produce substantially different results, making external validation and model comparison particularly important.

Response: We chose ERNIE-LSTM-TextCNN because ERNIE provides contextualized representations, the LSTM encodes sequence information, and TextCNN extracts local affective features. The model comparison completed during development is now reported on the same leakage-excluded 12,187-record labelled holdout: ERNIE-LSTM-TextCNN achieved accuracy and macro-F1 of 0.942 and MCC of 0.885, compared with character TF-IDF Multinomial Naive Bayes, character-count Bernoulli Naive Bayes, and a training-derived log-odds lexicon. The holdout is described as leakage-excluded rather than as an independently collected external corpus (Lines 245–258 and 379–381; Table S11).

Comment 9

__Reviewer comment:__ 9. Only the XGBoost model is used in the current analysis. Have the authors compared XGBoost with other commonly used machine-learning models to demonstrate its relative performance? In addition, how did the authors address and prevent overfitting, such as through cross-validation, hyperparameter tuning, or an independent test set? Were the data standardized or normalized before model training? How were the datasets divided into training, validation, and test sets? Based on the current description, the authors have not provided sufficient information to rule out the possibility of model overfitting.

Response: We now distinguish the primary citywide XGBoost analysis from the matched-support model comparison. The primary analysis uses 33,591 grids and 36 predictors; random-fold R^2 is 0.930 for the male-labelled model and 0.898 for the female-labelled model. The matched comparison uses the same 19,113 grids, 30 predictors, and random and buffered folds for Ridge, PLS regression, KNN, Linear SVR, and XGBoost. Every grid receives one out-of-fold prediction, test grids are excluded from fitting and preprocessing, and standardization for the conventional models is fitted only within each training fold. Model settings and the train–test separation are now stated explicitly (Lines 285–305 and 382–412; Table 3; Tables S12–S14; Figure S9).

Comment 10

__Reviewer comment:__ 10. There are too many subheadings in the Results section. I suggest consolidating them and organizing the section according to the main research findings, rather than dividing it into too many small subsections. This would improve the overall structure, readability, and logical coherence of the Results section.

Response: We consolidated the Results into four principal sections. Only the longest section, Global and local built-environment associations, contains two tertiary headings to separate global importance and nonlinear patterns from spatial contribution patterns. Repeated 5D statistics were removed from Section 3.4 and retained once in Section 3.3 (Lines 348–548).

Comment 11

__Reviewer comment:__ 11. The authors should provide a more detailed comparison between their findings and those reported in previous studies. In particular, they should explain the similarities and differences between their results and the existing literature and discuss the possible reasons for these discrepancies.

Response: We expanded the Discussion with direct, study-specific comparisons. Niu et al. provide a comparison for gendered spatial heterogeneity in geotagged urban emotion; Sun et al. and Gui et al. provide gender-specific comparisons involving green exposure, accessibility, commuting, and subjective well-being; Duan et al., Rui, and Zhang et al. provide Shanghai Weibo, Shenzhen settlement-context, and scale-dependent green-space comparisons. The Fuzhou village study is retained to distinguish shared nonlinear visual-environment patterns

from the stronger role of metropolitan population structure and centre–periphery organization in Shanghai (Lines 548–631).

Comment 12

> ***Reviewer comment:*** *12. The manuscript is overly lengthy and contains considerable redundant information. I suggest that the authors streamline the manuscript by integrating the key research methods and main findings, while moving less important methodological details and supplementary results to the Supporting Information (SI). In its current form, I do not think the manuscript is sufficiently concise, focused, or well organized to meet the standards for publication.*

Response: We streamlined the manuscript by retaining four Results sections, removing duplicate 5D statistics, and keeping the main findings in the article. Detailed predictor provenance, cleaning and grid-support summaries, sentiment and regression-model comparisons, model settings, and sensitivity results are now concentrated in the Supporting Information and Supplementary Data S1–S3 (Lines 348–548 and 710–719; Tables S1 and S9–S14).

Tables cited in the responses

Table V1. Minimum-user threshold sensitivity for the two platform-label groups.

Minimum users per group	Grids	Male posts	Female posts	Male user-grid incidences	Female user-grid incidences	Male R^2	Female R^2
1	4,832	182,653	317,450	117,472	259,792	0.846	0.937
3	2,752	171,960	310,000	112,786	255,123	0.793	0.905
5	1,988	165,293	304,101	109,263	251,081	0.783	0.894
10	1,258	154,263	293,716	103,757	243,824	0.727	0.866
20	779	140,591	280,374	96,611	233,630	0.718	0.842

Note. Counts are totals within the retained grids. R^2 is pooled out-of-fold XGBoost performance using the final area-level happiness outcomes.

Table V2. Regression-model performance on identical rows and predictors.

Outcome	Model	Random R^2	Random RMSE	Random MAE	Buffered R^2	Buffered RMSE	Buffered MAE
Male-labelled	Ridge	0.315	1.150	0.936	0.271	1.187	0.964
Male-labelled	PLS Regression	0.315	1.150	0.936	0.271	1.187	0.964
Male-labelled	KNN	0.417	1.061	0.851	0.290	1.171	0.946
Male-labelled	Linear SVR	0.315	1.150	0.938	0.271	1.186	0.965
**Male-labelled**	**XGBoost**	**0.568**	**0.914**	**0.714**	**0.363**	**1.109**	**0.884**
Female-labelled	Ridge	0.402	1.742	1.473	0.342	1.827	1.545
Female-labelled	PLS Regression	0.402	1.742	1.473	0.342	1.827	1.544
Female-labelled	KNN	0.449	1.672	1.420	0.310	1.871	1.598
Female-labelled	Linear SVR	0.402	1.742	1.477	0.343	1.826	1.547
**Female-labelled**	**XGBoost**	**0.587**	**1.448**	**1.156**	**0.353**	**1.812**	**1.482**

Note. Ridge regression, partial least squares (PLS) regression, K-nearest neighbours (KNN), linear support vector regression (Linear SVR), and XGBoost were compared during model development on the same 19,113 grids and 30 predictors. Buffered validation used 10 km spatial blocks with a 2.5 km exclusion buffer; values are pooled out-of-fold metrics. Bold identifies XGBoost, the model used for SHAP, PDP, and SVC analyses.

Table V3. Streetscape-support sensitivity for XGBoost.

Outcome	Support and predictor set	Grids	Predictors	Random R^2	Buffered R^2
Male-labelled	30 predictors; streetscape-supported	19,113	30	0.568	0.363
Male-labelled	23 non-streetscape; same grids	19,113	23	0.484	0.271
Male-labelled	23 non-streetscape; all grids	33,591	23	0.665	0.560
Female-labelled	30 predictors; streetscape-supported	19,113	30	0.587	0.353
Female-labelled	23 non-streetscape; same grids	19,113	23	0.523	0.281
Female-labelled	23 non-streetscape; all grids	33,591	23	0.510	0.209

Note. The final analytical table contained 14,478 grids with zero values for all eight streetscape indicators and 19,113 streetscape-supported grids. Zeros were not interpreted as verified environmental absence.

Table V4. Social-media data-processing flow supported by the retained records.

Stage	Retained records	Removed from preceding stage
Raw acquired geotagged posts	678,120	0
Initial retained records before text normalization	659,067	19,053
Text-normalized records	659,064	3
Exact cleaned-text deduplicated records	590,513	68,551
Final polarity-scored records with male/female platform label	507,707	82,806
Assigned to 500 m grid-count file	507,550	157 (0.03%)

Note. Only retained-record counts are tabulated. Before sentiment scoring, texts shorter than 5 or longer than 150 characters and emoji- or special-character-dominated texts without interpretable subjective emotional content were excluded; this rule has no separately recoverable stage count. Raw-file missing-gender and missing-text diagnostics may overlap. Grid-count linkage was a separate spatial-assignment step: 157 scored records fell outside Shanghai's municipal boundary after coordinate alignment and could not be assigned to a 500 m grid.

Table V5. Sentiment-model comparison on the common leakage-excluded holdout.

Model	Test n	Accuracy	Balanced accuracy	Macro-F1	MCC
**ERNIE-LSTM-TextCNN**	**12,187**	**0.942**	**0.942**	**0.942**	**0.885**
Character TF-IDF + MultinomialNB	12,187	0.883	0.883	0.883	0.767
Character count + BernoulliNB	12,187	0.925	0.925	0.925	0.850
Training-derived log-odds lexicon	12,187	0.893	0.893	0.893	0.786

Note. All models were evaluated on the same 12,187 labelled records. ERNIE-LSTM-TextCNN generated the continuous outcomes; the conventional baselines evaluate binary polarity classification only. Bold identifies the scoring model.

Table V6. Matched-support R^2 contrasts between XGBoost and conventional comparator models.

Outcome	Validation	Comparator	XGBoost R^2	Comparator R^2	ΔR^2
Male-labelled	Random five-fold	Ridge	0.568	0.315	0.253
Male-labelled	Random five-fold	PLS Regression	0.568	0.315	0.253
Male-labelled	Random five-fold	KNN	0.568	0.417	0.151
Male-labelled	Random five-fold	Linear SVR	0.568	0.315	0.253
Male-labelled	Buffered spatial	Ridge	0.363	0.271	0.092
Male-labelled	Buffered spatial	PLS Regression	0.363	0.271	0.092
Male-labelled	Buffered spatial	KNN	0.363	0.290	0.073
Male-labelled	Buffered spatial	Linear SVR	0.363	0.271	0.092
Female-labelled	Random five-fold	Ridge	0.587	0.402	0.185
Female-labelled	Random five-fold	PLS Regression	0.587	0.402	0.185
Female-labelled	Random five-fold	KNN	0.587	0.449	0.138
Female-labelled	Random five-fold	Linear SVR	0.587	0.402	0.185
Female-labelled	Buffered spatial	Ridge	0.353	0.342	0.010
Female-labelled	Buffered spatial	PLS Regression	0.353	0.342	0.010
Female-labelled	Buffered spatial	KNN	0.353	0.310	0.042
Female-labelled	Buffered spatial	Linear SVR	0.353	0.343	0.010

Note. ΔR^2 is XGBoost R^2 minus comparator R^2. All comparisons use the same 19,113 out-of-fold observations, predictor set, and fold definition within each validation scheme.
